# Global antimicrobial resistance: a system-wide comprehensive investigation using the Global One Health Index

**DOI:** 10.1186/s40249-022-01016-5

**Published:** 2022-08-23

**Authors:** Nan Zhou, Zile Cheng, Xiaoxi Zhang, Chao Lv, Chaoyi Guo, Haodong Liu, Ke Dong, Yan Zhang, Chang Liu, Yunfu Chang, Sheng Chen, Xiaokui Guo, Xiao-Nong Zhou, Min Li, Yongzhang Zhu

**Affiliations:** 1 Department of Animal Health and Food Safety, School of Global Health, Chinese Center for Tropical Diseases Research, Shanghai Jiao Tong University School of Medicine, Shanghai, 200025 China; 2 One Health Center, Shanghai Jiao Tong University-The University of Edinburgh, Shanghai, 200025 China; 3 National Institute of Parasitic Diseases at Chinese Center for Disease Control and Prevention (Chinese Center for Tropical Diseases Research), NHC Key Laboratory of Parasite and Vector Biology, WHO Collaborating Centre for Tropical Diseases, Shanghai, China; 4 Department of Population Medicine and Diagnostic Sciences, College of Veterinary Medicine, Cornell University, Ithaca, NY USA; 5 Department of Infectious Diseases and Public Health, Jockey Club College of Veterinary Medicine and Life Sciences, City University of Hong Kong, Kowloon, Hong Kong, China

**Keywords:** Global antimicrobial resistance, Global One Health Index, Antimicrobial resistance surveillance networks

## Abstract

**Background:**

Antimicrobial resistance (AMR) is one of the top ten global public health challenges. However, given the lack of a comprehensive assessment of worldwide AMR status, our objective is to develop a One Health-based system-wide evaluation tool on global AMR.

**Methods:**

We have further developed the three-hierarchical Global One Health Index (GOHI)-AMR indicator scheme, which consists of five key indicators, 17 indicators, and 49 sub-indicators, by incorporating 146 countries’ data from diverse authoritative databases, including WHO's *Global Antimicrobial Resistance and Use Surveillance System* (GLASS) and the European CDC. We investigated the overall- or sub-rankings of GOHI-AMR at the international/regional/national levels for data preprocessing and score calculation utilizing the existing GOHI methodology. Additionally, a correlation analysis was conducted between the GOHI-AMR and other socioeconomic factors.

**Results:**

The average GOHI-AMR score for 146 countries is 38.45. As expected, high-income countries (HICs) outperform the other three income groups on overall rankings and all five key indicators of GOHI-AMR, whereas low-income countries unexpectedly outperform upper-middle-income countries and lower-middle-income countries on the antibiotics-resistant key indicator (ARR) and ARR-subordinate indicators, including carbapenem-, β-lactam-, and quinolone resistance, and even HICs on aminoglycoside resistance. There were no significant differences among the four groups on the environmental-monitoring indicator (*P* > 0.05). GOHI-AMR was positively correlated with gross domestic product, life expectancy, and AMR-related publications, but negatively with natural growth rate and chronic respiratory disease. In contrast to Cyprus, the remarkably lower prevalence of "ESKAPE pathogens" in high-scoring Sweden and Denmark highlights Europe's huge gaps. China and Russia outperformed the other three BRICS countries on all key indicators, particularly India's ARR and Brazil's AMR laboratory network and coordination capacity. Furthermore, significant internal disparities in carbapenem-resistant *Klebsiella pneumoniae* (CRKP) and methicillin-resistant *Staphylococcus aureus* (MRSA) prevalence were observed between China and the USA, with MRSA prevalence both gradually declining, whereas CRKP prevalence has been declining in the USA but increasing in China, consistent with higher carbapenems-related indicator’ performance in USA.

**Conclusions:**

GOHI-AMR is the most comprehensive tool currently available for the assessment of AMR status worldwide. We discovered unique features impacting AMR in each country and offered precise recommendations to improve the capacity to tackle AMR in low-ranking countries.

**Graphical Abstract:**

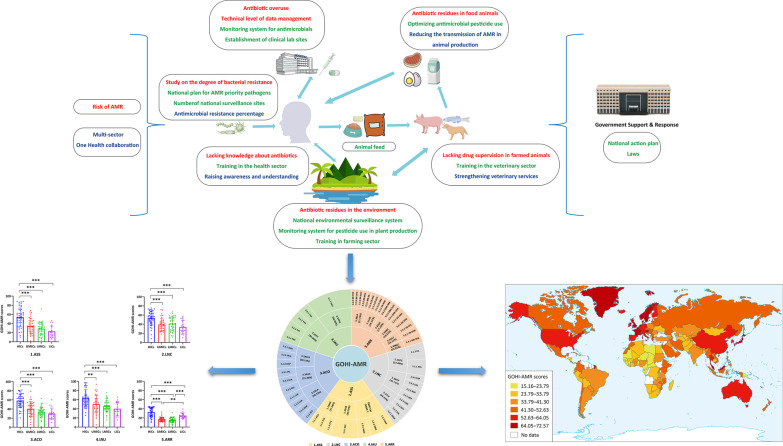

**Supplementary Information:**

The online version contains supplementary material available at 10.1186/s40249-022-01016-5.

## Background

Antimicrobial resistance (AMR), universally recognized as one of the most serious public health challenges of the twenty-first century [1], has grown into a global pandemic that poses a threat to human health and well-being, including health care, food production, and life expectancy [2]. According to the UK government's *Antimicrobial Resistance Review*, AMR would result in ten million annual deaths and a cumulative economic loss of GBP 100 trillion by 2050 [3, 4]. In addition, a comprehensive evaluation determined that bacterial AMR was responsible for 5 million human deaths worldwide in 2019 [5]. Furthermore, antimicrobials were estimated in food animal use at a global level of more than 130,000 tons in 2013 and 200,235 tons by 2030. Indeed, the United Nations (UN) reported that the increase in AMR was partly due to antimicrobial abuse in animals [6]. Meanwhile, resistant gene pools are frequently transferred into the surrounding environment once antibiotics are administered, including hospitals and animal farms [7]. The challenge with AMR is that it poses a significant risk to humans, animals, and even the environment [8]. Consequently, addressing AMR requires a multi-sectors or multi-systems strategy [9].

In 2015, the World Health Organization (WHO) launched a *Global Antimicrobial Resistance and Use Surveillance System* (GLASS), which collected, evaluated, and integrated data on AMR in humans across countries and territories. In addition, the Global Health Security Index (GHS Index), the first comprehensive evaluation of health security and related capacities, incorporates only five AMR indicators, including surveillance, detection, and reporting of AMR, as well as a national plan for AMR priority pathogens [10]. In 2016, the Food and Agriculture Organization (FAO), the World Organization for Animal Health (WOAH), and the WHO performed a tripartite AMR self-assessment country survey (TrACSS) to collect important data on the global AMR issue. However, none of the aforementioned approaches or databases provides a comprehensive analysis of the current global AMR situations under the One Health concept, nor a quantitative assessment index for integrating and comparing AMR data across countries, regions, and territories. Due to the close connection between AMR and humans, animals, and ecosystems, it is important to create and use a unique integrated multisectoral AMR evaluation approach that includes humans, terrestrial and aquatic animals, plants, and environments to investigate the current status of AMR worldwide.

The Global One Health Index (GOHI) systems have been built for the assessment of the One Health performance [11, 12]. It consists of three components in GOHI: external driver index (EDI), intrinsic driver index (IDI), and core driver index (CDI). AMR-related indicators are included in the CDI. In this study, we integrated many AMR-specific indicators into the GOHI framework, followed by building the GOHI-AMR database employing multi-source data from authoritative databases, such as GLASS and TrACSS. Based on the GOHI-AMR database, we can perform a comprehensive analysis of the current global AMR status and uncover major national-specific factors impacting AMR, by which we can identify the gaps in AMR at global and national levels so that the targeted improvement of AMR programs is feasible in time.

## Methods

### Collected AMR surveillance databases and index framework formulation.

**Fig. 1:**
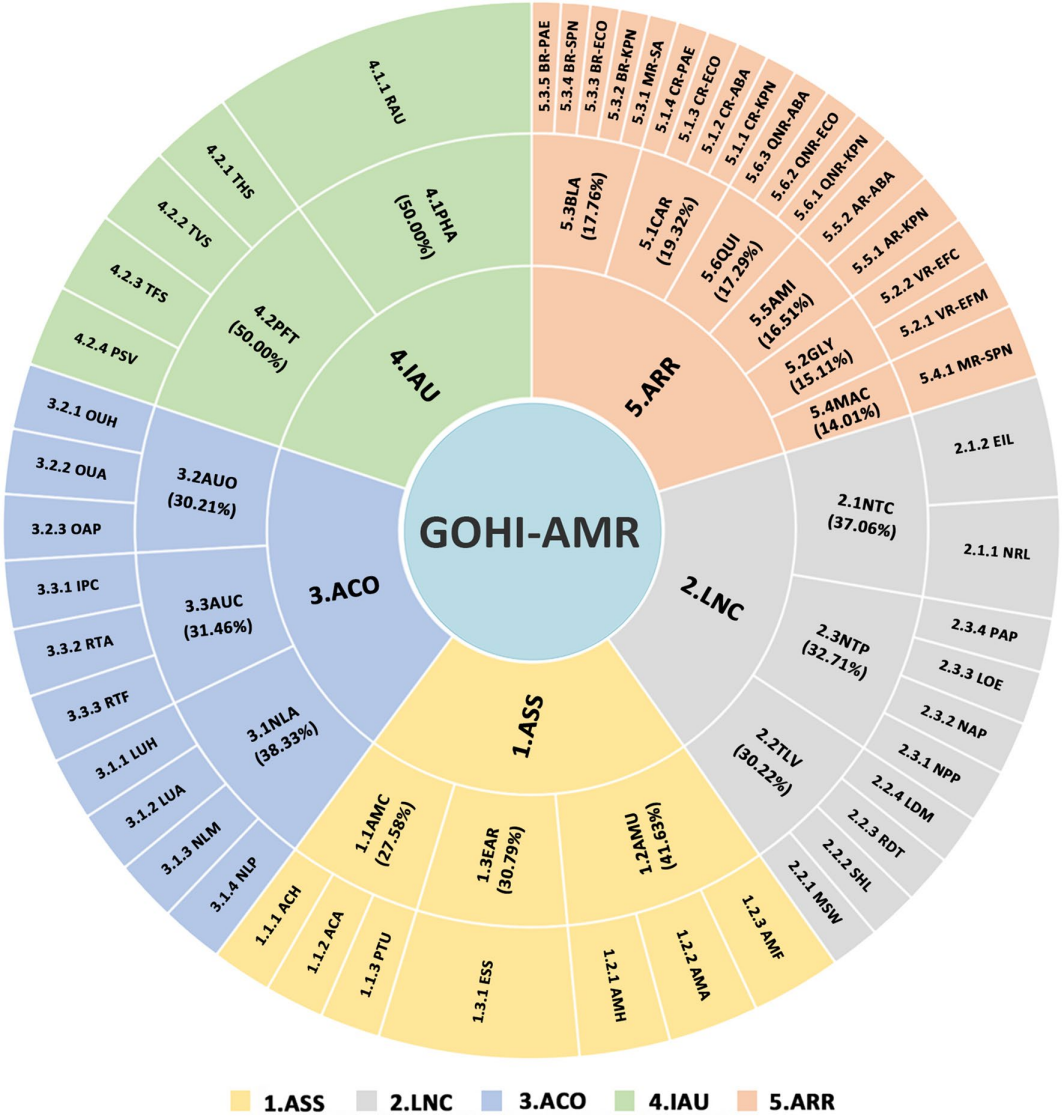
The entire framework and detailed weighted values for each of the GOHI-AMR structural indicators. The complete name and abbreviation of all indicators among three hierarchical indicators system of GOHI-AMR, consisting of 5 key indicators, 17 indicators, and 49 sub-indicators as following: 1. ASS (key indicator): AMR surveillance system. 1.1AMC (indicator): Antimicrobial consumption in both human and animals; 1.1.1 ACH (sub-indicator): Antimicrobial consumption in human; 1.1.2 ACA (sub-indicator): Antimicrobial consumption in animals; 1.1.3 PTU (sub-indicator): Pesticide Use; 1.2 AMU (indicator): Antimicrobial resistance status in human, animals, and food; 1.2.1 AMH (sub-indicator): AMR in human; 1.2.2AMA (sub-indicator): AMR in animals; 1.2.3 AMF: AMR in food; 1.3 EAR (indicator): Environmental surveillance system; 1.3.1 ESS: Environmental surveillance system; 2. LNC (key indicator): AMR laboratory network and coordination capacity. 2.1 NTC (indicator): National AMR capacity; 2.1.1 NRL (sub-indicator): National reference laboratory; 2.1.2 EIL (sub-indicator): Effective integration of laboratories; 2.2 TLV (indicator): Technical promotion score in AMR; 2.2.1 MSW (sub-indicator): multi-sector working on AMR; 2.2.2 SHL (sub-indicator): Standardization and harmonization of laboratories; 2.2.3 RDT: Relevance of diagnostic techniques; 2.2.4 LDM (sub-indicator): Technical level of data management; 2.3 NTP (indicator): National action plan formulations; 2.3.1 NPP (sub-indicator): National plan for AMR priority pathogens; 2.3.2 NAP (sub-indicator): National action plan on AMR; 2.3.3 LOE (sub-indicator): National action plan on AMR linked to any other existing action plans; 2.3.4 PAP (sub-indicator): Publishment of action plan; 3. ACO (key indicator): Antimicrobial control and optimization. 3.1 NLA (indicator): National law(s) for antibiotic use; 3.1.1 LUH (sub-indicator): National law(s) for the use of antibiotics in humans; 3.1.2. LUA (sub-indicator): National law(s) for antibiotic use in animals; 3.1.3. NLM: (sub-indicator) National law(s) on marketing of pesticides; 3.1.4. NLP (sub-indicator): National law(s) on prohibits the use of antibiotics; 3.2 AUO (indicator): Optimization of antimicrobial use; 3.2.1 OUH (sub-indicator): Optimizing antimicrobial use in human health; 3.2.2 OUA (sub-indicator): Optimizing antimicrobial use in animal health; 3.2.3 OAP (sub-indicator): Optimizing antimicrobial pesticide use in plants; 3.3 AUC (indicator): Interruption capacity of antimicrobial resistance transmission; 3.3.1 IPC (sub-indicator): Infection Prevention and Control in human; 3.3.2 RTA (sub-indicator): Reduce transmission of AMR in animal production; 3.3.3 RTF (sub-indicator): Reduce transmission of AMR in food processing; 4.IAU (key indicator): Improve awareness and understanding; 4.1PHA (indicator): Raising awareness and understanding; 4.1.1 RAU (sub-indicator): Raising awareness and understanding; 4.2 PFT (indicator): Professional training activities in multi-sectors; 4.2.1 THS (sub-indicator): Training in the human health sector; 4.2.2 TVS (sub-indicator): Training in the veterinary sector; 4.2.3TFS (sub-indicator): Training in the farming sector; 4.2.4 PSV (sub-indicator): Progress with strengthening veterinary services; 5.ARR (key indicator): Antimicrobial resistance rate for important antibiotics. 5.1 CAR (indicator): Carbapenems-resistents for multi-specise; 5.1.1 CR-KPN (sub-indicator): Carbapenems-resistent *Klebsiella pneumoniae*; 5.1.2 CR-ABA (sub-indicator): Carbapenems-resistent *Acinetobacter baumannii*; 5.1.3 CR-ECO (sub-indicator): Carbapenems-resistent *Escherichia coli*; 5.1.4CR-PAE (sub-indicator): Carbapenems-resistent *Pseudomonas aeruginosa*; 5.2 GLY (indicator): Vancomycin-resistents for *Enterococcus faecium*, and *Enterococcus faecalis*; 5.2.1 VR-EFM (sub-indicator): Vancomycin-resistant *Enterococcus faecium*; 5.2.2 VR-EFC (sub-indicator): Vancomycin-resistant *Enterococcus faecalis*; 5.3 BLA (indicator): β-lactams-resistants for multi-specises; 5.3.1 MR-SA (sub-indicator): Methicillin-resistant *Staphylococcus aureus*; 5.3.2 BR-KPN (sub-indicator): Third-generation β-lactams-resistent *Klebsiella pneumoniae*; 5.3.3 BR-ECO (sub-indicator): Third-generation β-lactams-resistent *Escherichia coli*; 5.3.4 BR-SPN (sub-indicator): Third-generation β-lactams-resistent *Streptococcus pneumoniae*; 5.3.5 BR-PAE (sub-indicator): Third-generation β-lactams-resistent *Pseudomonas aeruginosa*; 5.4 MAC (indicator): Macrolides-resistent for *Streptococcus pneumoniae*; 5.4.1 MR-SPN (sub-indicator): Macrolides-resistent *Streptococcus pneumoniae*; 5.5 AMI (indicator): Aminoglycosides-resistents for *Klebsiella pneumoniae* and *Acinetobacter baumannii*; 5.5.1 AR-KPN (sub-indicator): Aminoglycosides-resistent *Klebsiella pneumoniae*; 5.5.2AR-ABA (sub-indicator): Aminoglycosides-resistent *Acinetobacter baumannii*; 5.6 QUI (indicator): Quinolone-resistents for *Klebsiella pneumoniae*, *Escherichia coli*, *Acinetobacter baumannii*; 5.6.1QNR-KPN (sub-indicator): Quinolone-resistant *Klebsiella pneumoniae*; 5.6.2QNR-ECO (sub-indicator): Quinolone-resistant *Escherichia coli*; 5.6.3QNR-ABA (sub-indicator): Quinolone-resistant *Acinetobacter baumannii*

All AMR data is obtained from multiple global authoritative online databases such as WHO, FAO, GHS, and WOAH. The GLASS of the WHO, GHS, TrACSS [13–15], and several other currently authoritative databases associated with national or continental AMR monitoring, such as the European Centre for Disease Prevention and Control (ECDC), the Centers for Disease Control and Prevention (CDC) of the United States of America (USA), and the China Antimicrobial Resistance Surveillance System (CARSS) in China [16–18], were eventually chosen. The aforementioned databases were used to acquire AMR-related data from 220 countries globally. Meanwhile, after the deletion of 74 countries, 146 countries with complete data remained (detailed data in Additional file 1: Table S1). We excluded duplicated identical issues and preferred actionable indicators among these AMR databases. In addition to the aforementioned qualitative criteria, we further conducted a PubMed search using the terms "antimicrobial resistance" and a large number of well-known drug-resistant bacteria. Based on the search results, we newly added the quantitative prevalence of multiple bacteria-antibacterial drug combinations that are of great concern to researchers worldwide and pose a major threat to global human health in this study. As seen in Fig. 1, 4 of 5 GHS indicators, 1 of 10 GLASS indicators, 27 of 48 TrACSS indicators, and 17 new indicators from AMR monitoring data were incorporated into the GOHI-AMR.

### Construction of the three-level GOHI-AMR indicator system

We refer to the existing AMR evaluation indicator frameworks from the known authoritative databases mentioned above. In AMR-related publications, the emergence and dissemination of drug-resistant bacteria is comprehensive estimated. Here, we found the impact of five aspects that are crucial for AMR, including antimicrobial consumption monitoring [19], multi-sector cooperation, prevention and control of AMR, being well-informed about AMR in the current community, and the prevalence of key AMR bacteria within qualitative characteristics. Finally, based on their logical classification and expert’s consensus, we categorized the GOHI-AMR system into the following five key indicators, totally containing 17 indicators and 49 sub-indicators:

(i) ASS (AMR surveillance system) consists of three indicators and seven sub-indicators [20]; (ii) LNC (AMR laboratory network and coordination capacity) consists of three indicators and ten sub-indicators [21]; (iii) ACO (Antimicrobial control and optimization) consists of three indicators and ten sub-indicators [22]; (iv) IAU (Improve awareness and understanding) consists of two indicators and five sub-indicators [23]; (v) ARR (Antimicrobial resistance rate for important antibiotics) consists of six indicators and 17 sub-indicators [24–27] (the detailed information in Additional file 2: Table S2).

### The distinct weighted value of each indicator determined by the GOHI-developed algorithm

Then, we performed several rounds of expert committee surveys on all indicators and estimated each indicator's varying weight value using the fuzzy analytic hierarchy process (FAHP) algorithm developed by GOHI. We defined our exclusion criteria for each metric and country/territory based on the missing data rate, as Zhang et al. recently published [11, 12]. Our analysis excluded these indicators with missing data in over 160 countries or nations/territories with a missing data rate of more than 50%. We interpolate missing values for the included variables by averaging the sociodemographic characteristics of the three closest equivalents. Overly polarized values have been fixed by picking a random number from the normal distributions N (0, 0.16^2^) (for value 0) and N (1, 0.16^2^) (for value 1).

The following equation is used to determine the normalized score of an indicator:

$${N}_{i}=\left\{\frac{\begin{array}{c}0\\ {X}_{i}-{X}_{worst}\end{array}}{\begin{array}{c}{X}_{best}-{X}_{worst}\\ 100\end{array}}\times 100\right.,$$

where *N*_*i*_ represents the normalized indicator score for the i-th country; *X*_*i*_ denotes the original indicator value of the i-th country; *X*_*best*_ denotes the indicator's best value, and *X*_*worst*_ denotes the indicator's worst value.

$${US}_{i}=\sum_{1}^{m}{LS}_{\mathrm{ij}}\times {W}_{\mathrm{j}}, \sum_{1}^{\mathrm{m}}{W}_{\mathrm{j}}=1.$$

The scores for the upper-level indicators were calculated as the weighted sum of the scores for the lower-level indicators: *US*_*i*_ is the score for the i-th country's upper-level indicators; *LS*_*ij*_ is the score for the i-th country's j-th lower-level indicators; m is the total number of lower-level indicators below the upper-level indicator, and *W*_*j*_ is the weight of the j-th lower-level indicator.

### Correlation analysis between GOHI-AMR and 9 additional external factors

In order to investigates the relationship between GOHI-AMR and nine additional external factors, including five socioeconomic factors [gross domestic product (GDP) per capita, gross national income (GNI) per capita, domestic health expenditure, population density, and natural growth rate], life expectancy and chronic respiratory disease, one environmental forest area [28–30], and the total number of PubMed publications related to One Health and AMR in different countries, we performed the correlation analysis following the Spearman’s rank method. The *r* > 0.5 or <  − 0.5 with *P* < 0.05 were statistically significant. Statistical analyses were performed using GraphPad Prism version 9.0.1 (GraphPad Software, LLC., USA) and SPSS version 21.0 (SPSS, IBM; Inc., USA).

## Results

### Global distribution of GOHI-AMR overall scores

**Fig. 2:**
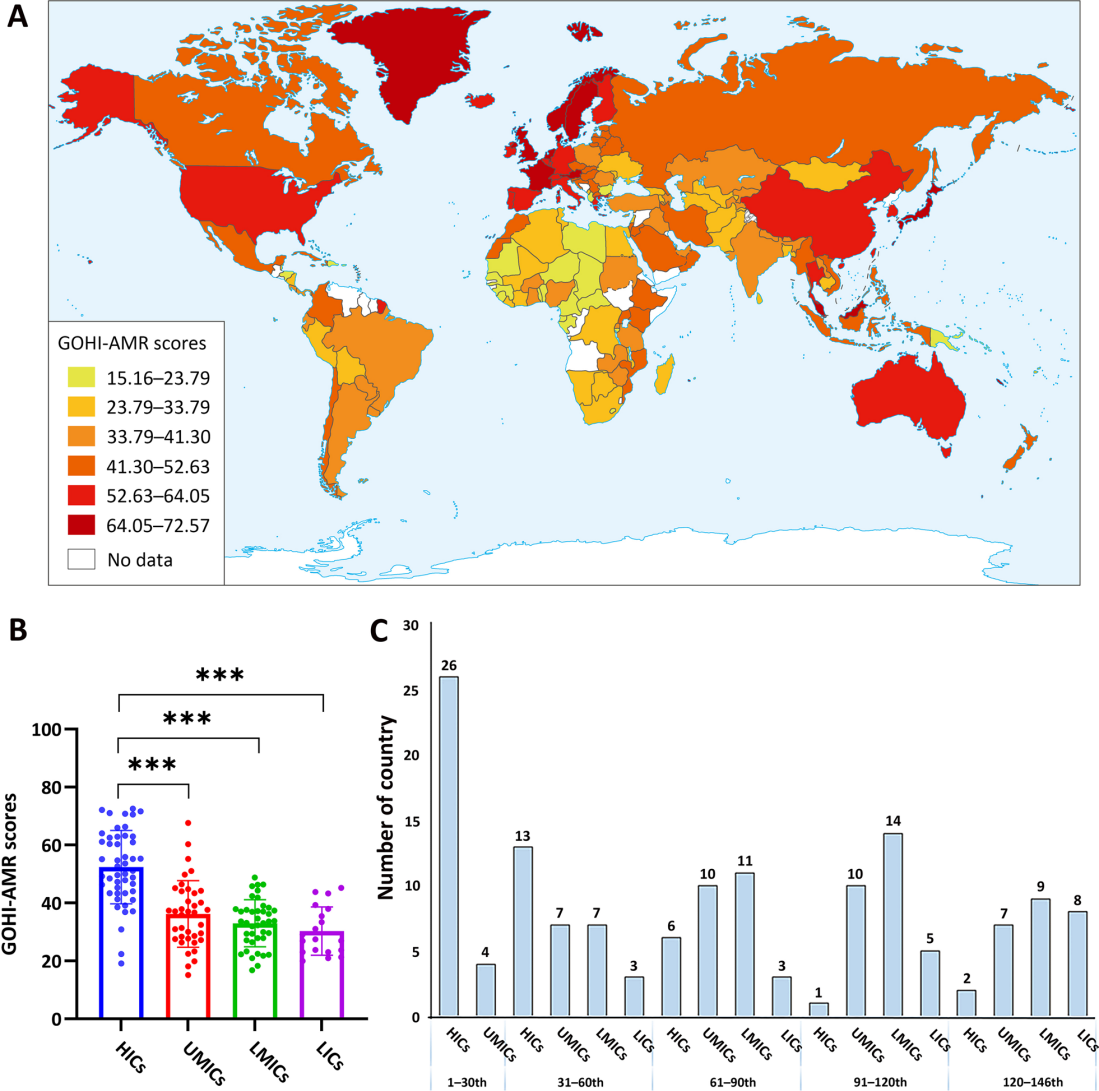
The summarized global GOHI-AMR scores among the four income nation groups. **A** A worldwide distribution map of the GOHI-AMR overall scores. **B** A statistical analysis of the GOHI-AMR scores in each of the four income groups. **C** Distribution of overall GOHI-AMR scores among four groupings of high-income countries. *GOHI-AMR* Antimicrobial resistance in Global One Health Index, *HICs* high-income countries, *UMICs* upper-middle-income countries, *LMICs* lower-middle-income countries, *LICs* low-income countries

The World Bank categorized the 146 countries into four income nation groups, including 48 high-income countries (HICs), 38 upper-middle-income countries (UMICs), 41 low-middle-income countries (LMICs), and 19 low-income countries (LICs) [31]. As shown in Fig. 2A, B, the average GOHI-AMR score globally is 39.85. HICs had substantially higher GOHI-AMR overall scores [mean: 52.35 ± 12.68, interquartile range (IQR) = 19.21] than UMICs (36.23 ± 11.51, IQR = 16.40), LMICs (32.99 ± 8.12, IQR = 10.23), and LICs (30.30 ± 8.31, IQR = 14.89) (*P* < 0.001). Except for four UMICs (Malaysia, Thailand, China, and Belarus), the top 30 highest-scoring countries are mostly HICs, like France (overall score: 72.57, overall ranking: 1st/146), Sweden (72.10, 2nd/146), and Norway (71.63, 3rd/146). The bottom 20 countries are all LMICs and LICs, like Cameroon (20.96, 139th/146) and Niger (19.97, 140th/146). Surprisingly, several HICs and UMICs, such as Seychelles (19.13, 142nd/146), Gabon (15.16, 146th/146), and Albania (22.43, 132nd/146), were also found in the ten countries with the lowest scores. This shows that income or economic development was not the only key factor affecting AMR in these countries (see Additional file 2: Table S3 for more information).

### Key indicators of the GOHI-AMR among the four income nation groups

**Fig. 3:**
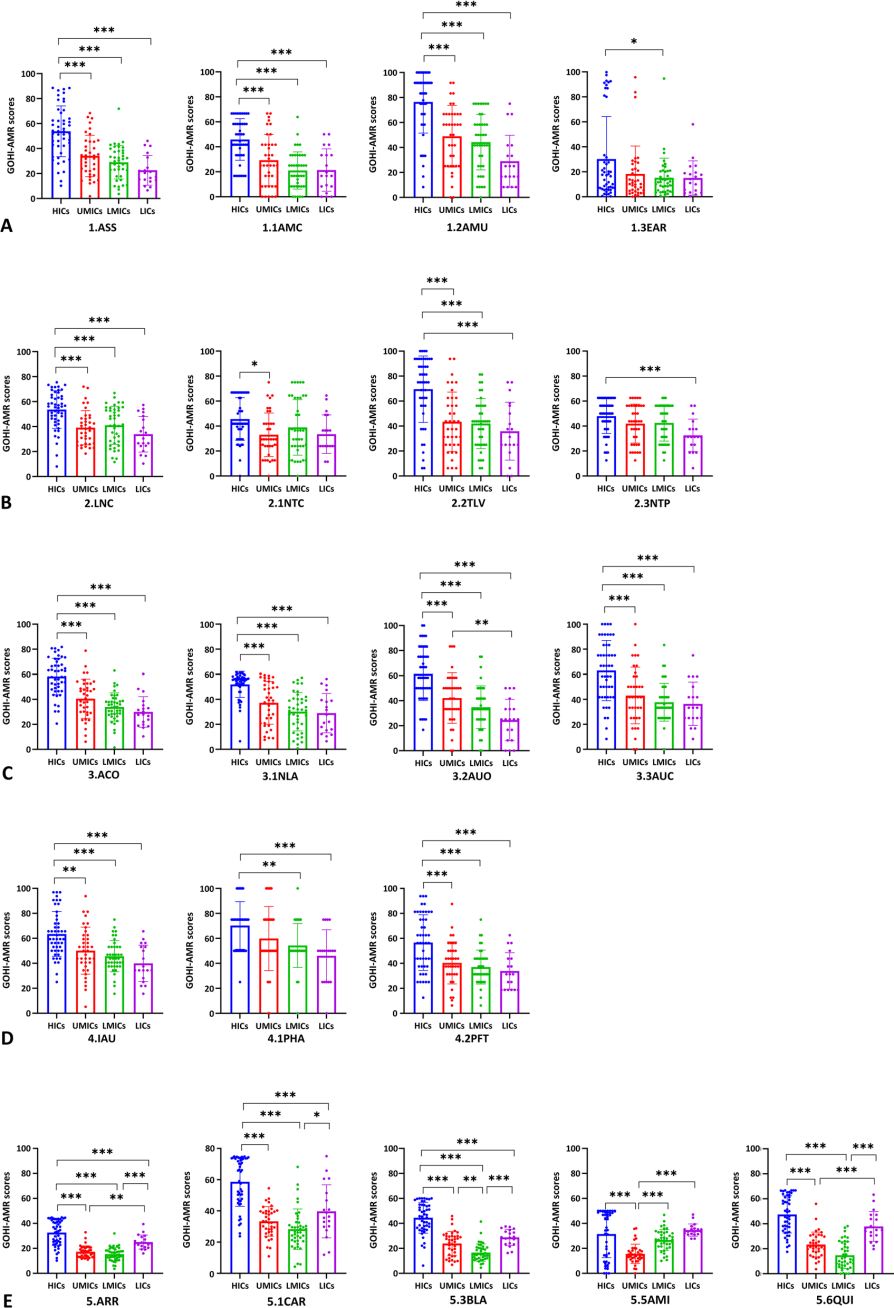
The GOHI-AMR scores of the five key indicators and seventeen indicators among the four income nation groups.** A** GOHI-AMR scores of the quantitative indicators in the sub-indicator CAR among the four income nation groups;** B** GOHI-AMR scores of the quantitative indicators in the sub-indicator BLA among the four income nation groups;** C** GOHI-AMR scores of the quantitative indicators in the sub-indicator AMI among the four income nation groups;** D** GOHI-AMR scores of the quantitative indicators in the sub-indicator QUI among the four income nation groups. * represnts *P* value < 0.05, ** *P *value < 0.01, *** *P* value < 0.001. The complete names of the indicators are listed in the notes of Fig. 1

In addition, we analyzed each key indicator across income-based national groups. In Fig. 3A–E, HICs performed better than the other three groups on all five key indicators (*P* < 0.001), whereas LICs unexpectedly outperformed UMICs and LMICs on the key indicator ARR (*P* < 0.001). Interestingly, we further discovered that LICs performed better than UMICs and LMICs on the indicators, carbapenem resistance (CAR), β-lactam resistance (BLA), and quinolone resistance (QUI), and even outperformed HICs on the indicator aminoglycoside resistance (AMI). Simultaneously, HICs outperformed UMICs only on AMI, with no difference between HICs, LMICs, and LICs. Surprisingly, there were no significant differences between the four groups on the indicators EAR (environmental surveillance system), 2.1NTC (national AMR capability), or NTP (national action plan formulations). Only eight countries, including the Netherlands, Austria, and Australia from HICs, Jordan from UMICs, and Vietnam from LMICs, scored more than 90 on the EAR. The remaining 130 countries all scored less than 40, which shows that national environmental surveillance networks need to be set up as soon as possible.

**Fig. 4:**
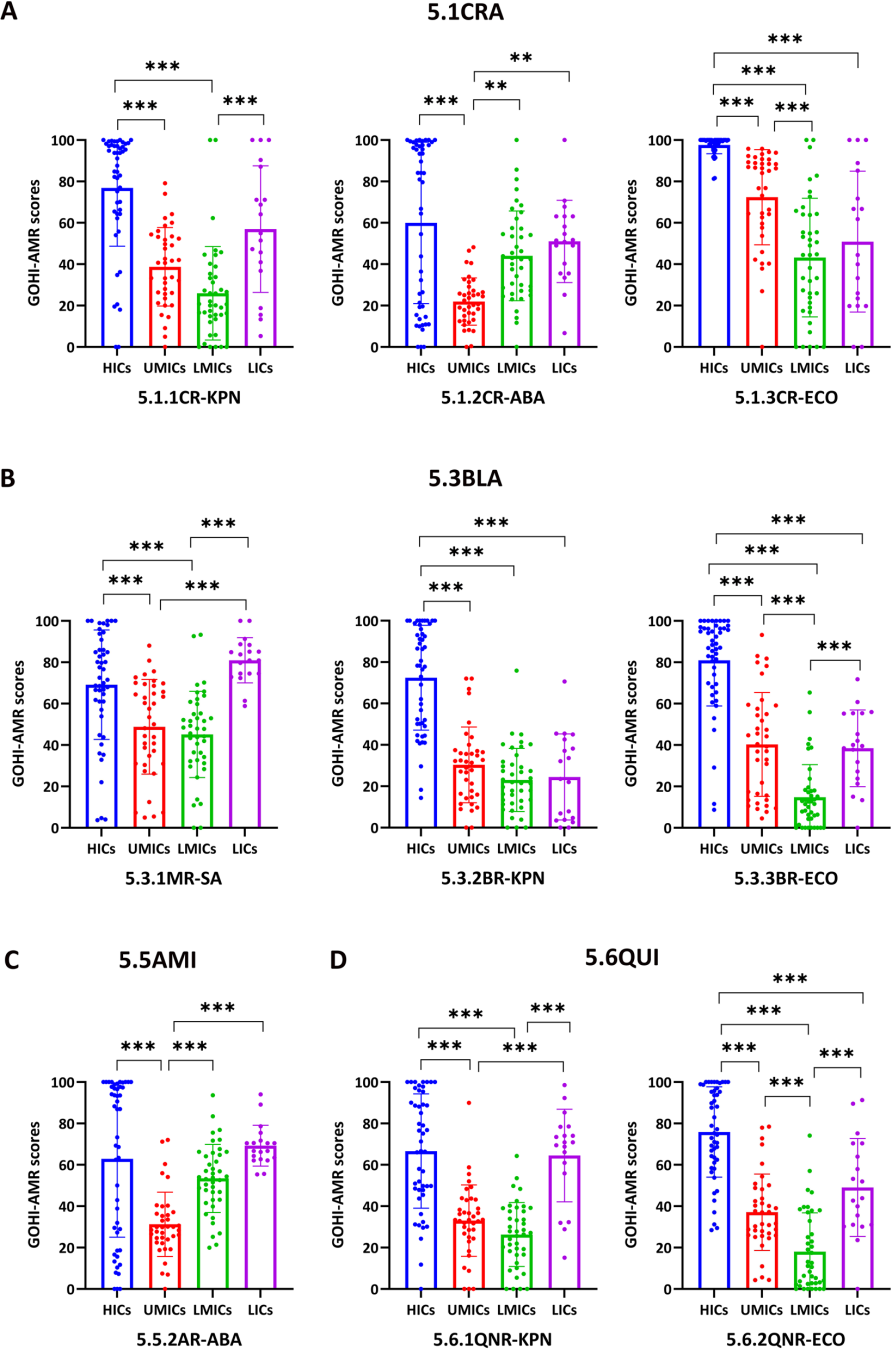
GOHI-AMR Scores of the quantitative indicators and sub-indicators in the key indicator ARR among the four income nation groups. Certain indicators have been omitted because the required data are unavailable in more than 160 nations, or fewer than half of all countries. * represnts *P* value < 0.05, ** *P* value < 0.01, *** *P* value < 0.001

In this study, the ARR is a unique quantitative key indicator encompassing many sub-indicators. Figure 4 demonstrates that HICs differ considerably from the other three groups in the CAR (*P* < 0.001). In the sub-indicator CR-ABA (carbapenem-resistant *Acinetobacter baumannii*), HICs, LMICs, and LICs scored substantially higher than UMICs (*P* < 0.001). In CR-ECO (carbapenem-resistant *Escherichia coli*), its scores in LMICs and LICs were higher than that in UMICs. Moreover, HICs and LICs outperformed UMICs and LMICs in the two carbapenem-resistant sub-indicators (CR-ABA and CR-ECO). Surprisingly, in the MR-SA (methicillin-resistant *Staphylococcus aureus*, MRSA), LICs scored even much higher than HICs, suggesting a distinct AMR epidemic pattern different from *Klebsiella pneumoniae* and *E. coli*. Between HICs and LICs, there were no significant differences in the aminoglycoside-resistant and quinolone-resistant indicators (AMI and QUI), and both groups scored higher than UMICs and LMICs. HICs and LICs had considerably higher sub-scores than UMICs and LMICs, especially for the sub-indicator QNR-KPN (quinolone-resistant *K. pneumoniae*). As expected, HICs continue to do better than the other three income groups in most antimicrobial-bacteria combinations. However, AMR is widespread in most UMICs and LMICs, in part because of the high use of antimicrobials during rapid economic growth (all the indicator scores are in Additional file 2: Table S4).

### Correlation between GOHI-AMR and nine external factors

**Fig. 5:**
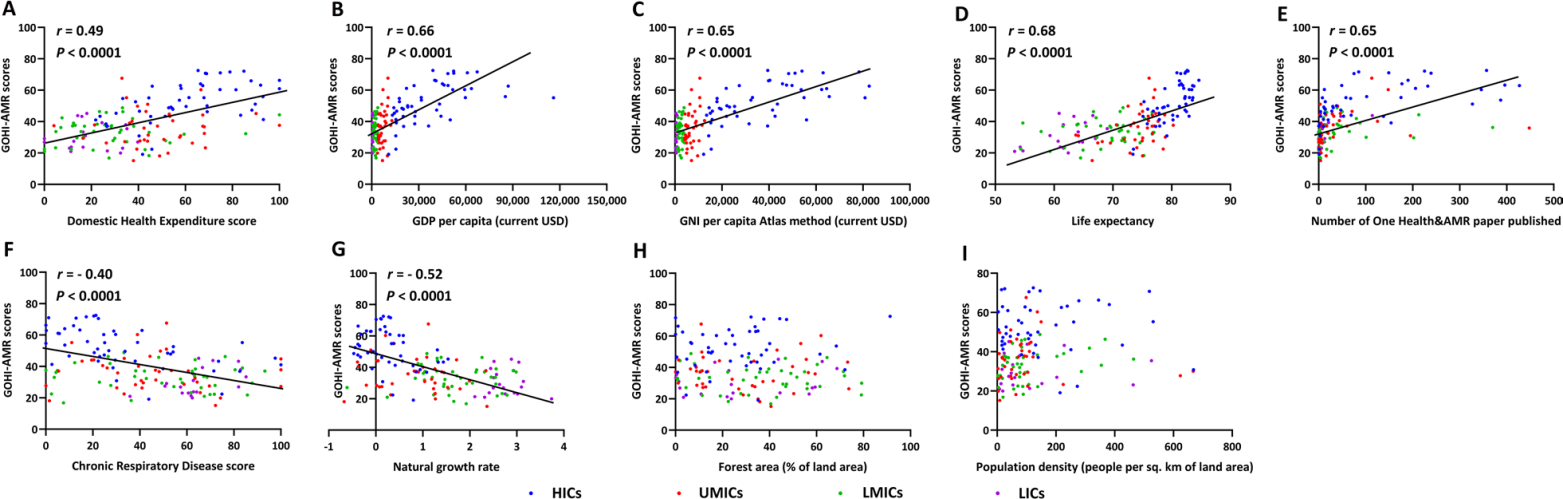
Correlation between several external variables and GOHI-AMR total scores. **A** Domestic Health Expenditure Scores; **B** GDP per capita (current USD); **C** GNI per capita Atlas method (current USD); **D** Life expectancy; **E** Number of One health & AMR publications; **F** Chronic Respiratory Disease Score; **G** Natural growth rates; **H** Forest area (% of land area); **I** Population density (people per sq.km of land area). *GOHI-AMR* Antimicrobial resistance in Global One Health Index, *HICs* high-income countries, *UMICs* upper-middle-income countries, *LMICs* lower-middle-income countries, *LICs* low-income countries

In fact, it is well-known that the status and governance capability of antimicrobial resistance should be certainly related to a country's socioeconomic, environmental, medical, and health achievements, as well as its scientific research. Therefore, we chosen nine external key factors from other GOHI and World Bank indicators for correlation analysis. The correlation analysis results between GOHI-AMR and nine additional external factors, were shown in Fig 5A–I. There was a statistically significant positive correlation between GOHI-AMR scores and GDP per capita (*r* = 0.66, *P* < 0.0001), GNI per capita (*r* = 0.65, *P* < 0.0001), life expectancy (*r* = 0.68, *P* < 0.0001), and the number of PubMed publications on One Health & AMR (*r* = 0.65, *P* < 0.0001) (Fig. 5A–E). Simultaneously, a statistically significant negative correlation between GOHI-AMR scores and natural growth rates (*r* = − 0.52, *P* < 0.0001) was discovered (Fig. 5F, G). Finally, no correlation was established between GOHI-AMR and population density or forest area (Fig.  5H, I).

As depicted in Fig. 5A–C, the majority of HICs and some UMICs with high overall rankings in GOHI-AMR undoubtedly have greater economic and health investments. The majority of LICs and LMICs lag in economic development due to their inability to manage AMR effectively. Simultaneously, the natural growth rates of most developed countries are often lower than those of the economically underdeveloped countries or territories. Certainly, the natural growth rate is negatively related to the GOHI-AMR scores, drug-resistant bacteria pose a grave threat to human life. Consequently, each country's GOHI-AMR score has a considerable impact on the life expectancy of its population. Also, these countries published more articles about One Health and AMR. This made their GOHI-AMR scores higher, showing that national research is a key part of putting the One Health strategy against AMR into action.

### Analysis of differential GOHI-AMR scores throughout Europe

Regarding the majority of HICs located in Europe, we performed the differentiation analysis on GOHI-AMR in Europe. A set of long-term AMR surveillance data was collected from the ECDC on the important antimicrobial bacteria ESKAPE, encompassing *Enterococcus faecium*, *Staphylococcus aureus*, *Klebsiella pneumoniae*, *Acinetobacter baumannii*, *Pseudomonas aeruginosa*, and other *Enterobacter* species, and examined the possible correlation between GOHI-AMR scores and the actual AMR prevalence of ESKAPE [32].

**Fig. 6:**
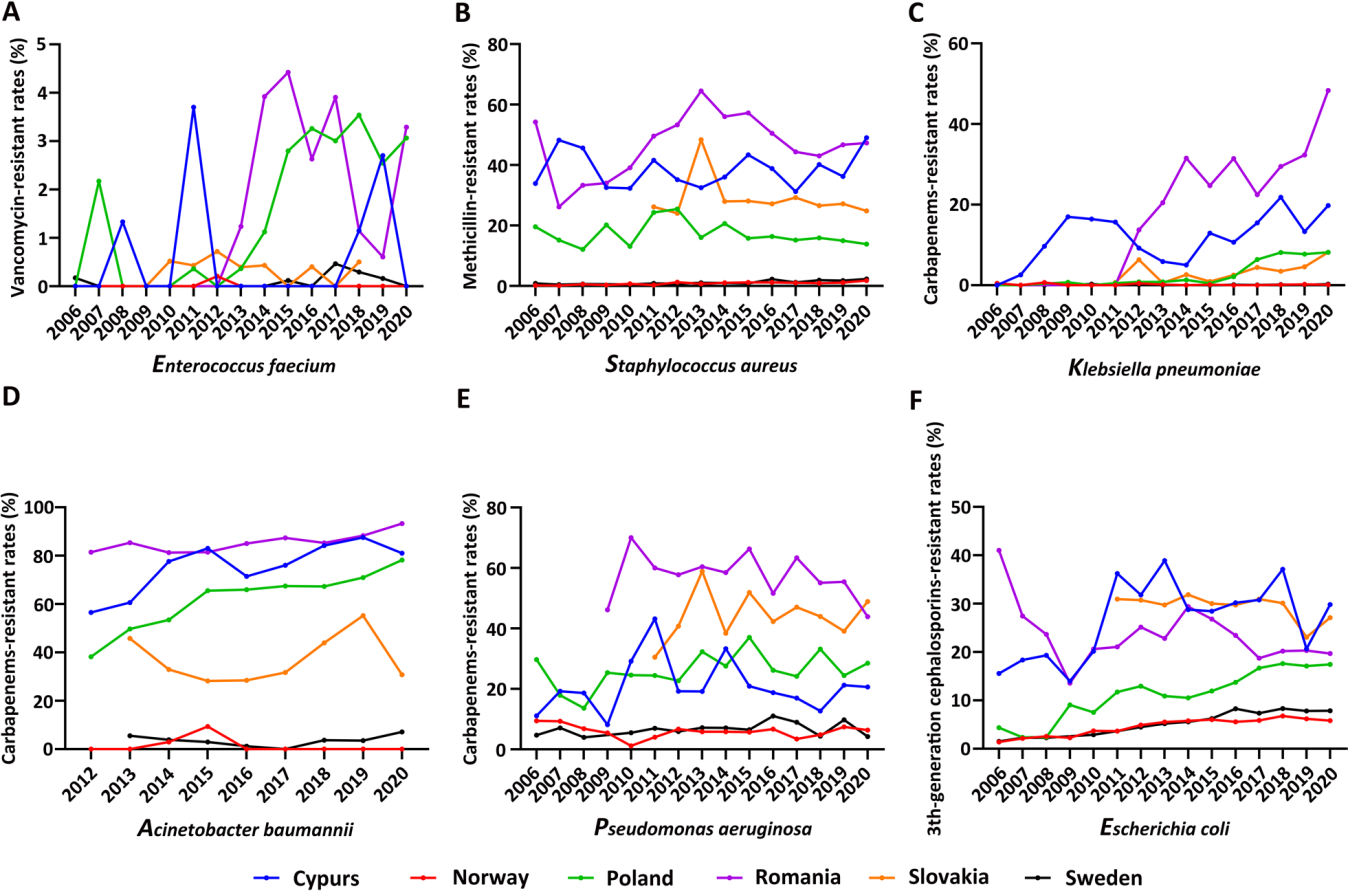
The actual prevalent rates of ESKAPE across Europe from 2006 to 2020. **A ***Enterococcus faecalis*; **B** *Staphylococcus aureus*; **C** *Klebsiella pneumoniae*; **D** *Acinetobacter baumannii*; **E** *Pseudomonas aeruginosa*; **F** *Escherichia coli*

Based on the GOHI-AMR overall ranking, we selected two European countries with high overall scores (Sweden and Norway) and four European countries with relatively low scores (Cyprus, Poland, Slovakia, and Romania). The key indicators of ARR’s scores are depicted in Fig. 6. Norway (score 44.81, ranked 1st/146) and Sweden (45.00, ranked 6th/146) scored significantly higher than Cyprus (15.42, 107th/146), Poland (21.17, 67th/146), Slovakia (27.73, 38th/146), and Romania (13.48, 118th/146). This shows that there are huge differences in common AMR prevalence and their control outcomes in the six European countries. Undoubtedly, both AMR prevalence and control outcomes in Sweden and Norway have consistently outperformed those of the other four European countries. The most notable difference between the two groups, as seen in Fig. 6D, was in the sub-indicator CR-ABA. The sub-scores of CR-ABA in Norway (100.00), Sweden (93.47), and Slovakia (66.80) are all sharply higher than those in Poland (13.46) and Cyprus (10.31). These findings show that even among these European HICs, there are big differences in AMR, especially in Cyprus. This means that economic growth may not be the most important factor in impacting AMR.

### The GOHI-AMR overall ranking among the BRICS countries

The GOHI-AMR rankings of the BRICS countries have significant implications for the majority of LMICs, as well as a few LICs and UMICs. The average GOHI-AMR overall score of the BRICS countries is 41.60. China (overall score: 55.21; overall ranking: 23rd/146) and Russia (49.79, 31st/146) performed significantly better than India (36.23, 87th/146), Brazil (35.86, 88th/146), and South Africa (30.93, 103rd/146).

**Fig. 7:**
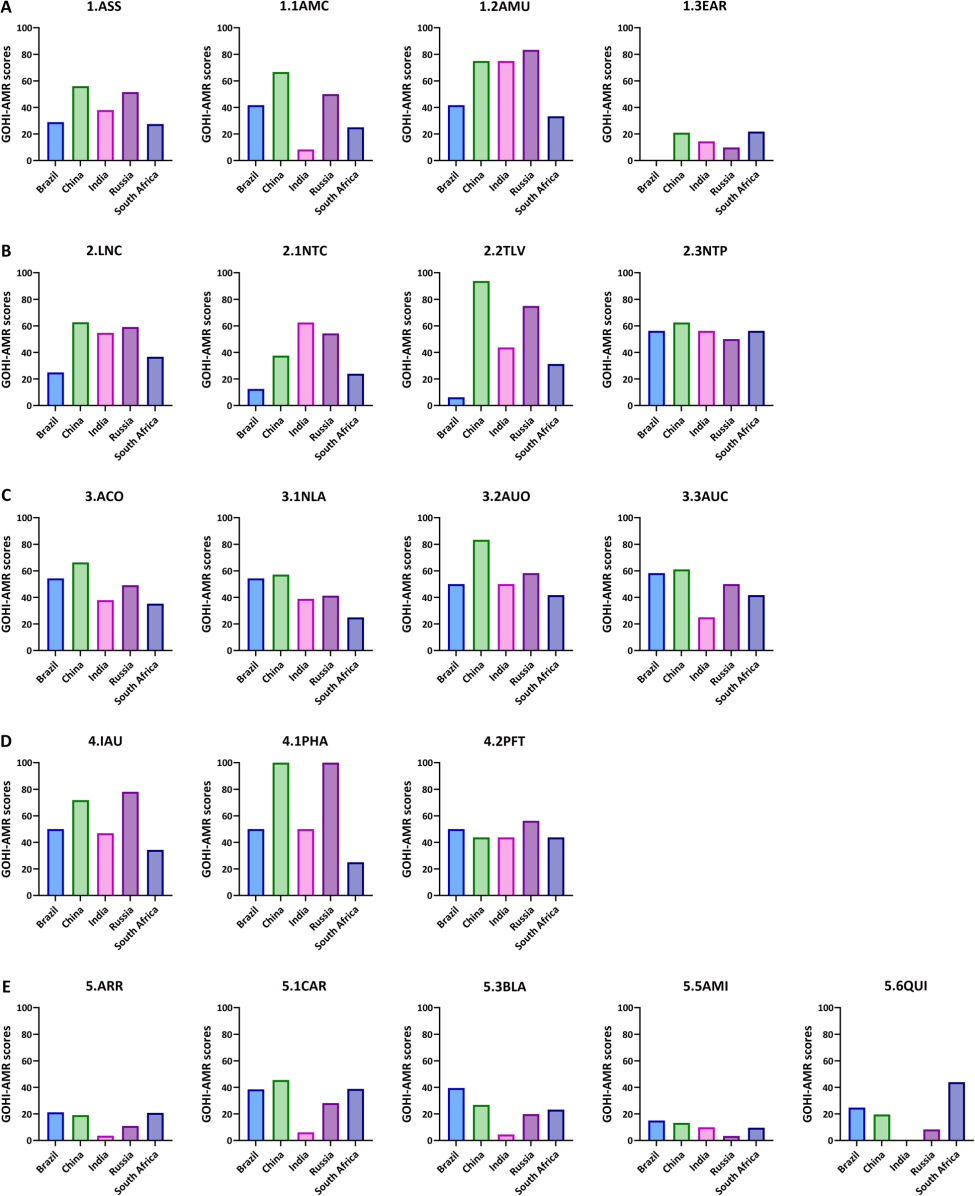
The GOHI-AMR sub-scores of the 5 key indicators and 17 indicators among the BRICS. **A** GOHI-AMR scores of the key indicator ASS and sub-indicators among the BRICS;** B** GOHI-AMR scores of the key indicator LNC and sub-indicators among the BRICS;** C** GOHI-AMR scores of the key indicator ACO and sub-indicators among the BRICS;** D** GOHI-AMR scores of the key indicator IAU and sub-indicators among the BRICS;** E** GOHI-AMR scores of the key indicator RR and sub-indicators among the BRICS. The complete names of the indicators are listed in the notes of Fig. 1

As expected, the BRICS countries demonstrate some variation in their GOHI-AMR indicators in Fig. 7. China and Russia ranked in the top 20% of the 146 countries, while most indicators ranked in the top 30%, except the ARR in China (score: 19.13, ranking: 82nd/146) and Russia (10.99, 136th/146), and the ACO in Russia (49.17, 51st/146). Both China and Russia have highly prevalent rates of AMR bacteria, such as high resistance in the indicators AMI (aminoglycoside) and QUI (quinolone). Meanwhile, Russia was not doing well in comprehensive antimicrobial control and optimization efforts, such as national legislation controlling antibiotic use in livestock and pesticide marketing, corresponding with its lower scores in NLA. On the other hand, the 15th ranking of the key indicator IAU in Russia showed substantial improvement in the Russian population's knowledge of AMR. Simultaneously, India and Brazil each have some deficiencies, with the most notable being India's ARR (score: 3.63, ranking: 146th/146) and Brazil's LNC (24.92, 123rd/146). Surprisingly, the scores of all five key indicators in South Africa were far below the global average. The other three BRICS countries performed poorly compared to China and Russia's advantaged key indicators, such as ASS and IAU.

### MRSA and CRKP between China and the USA based on GOHI-AMR

**Fig. 8:**
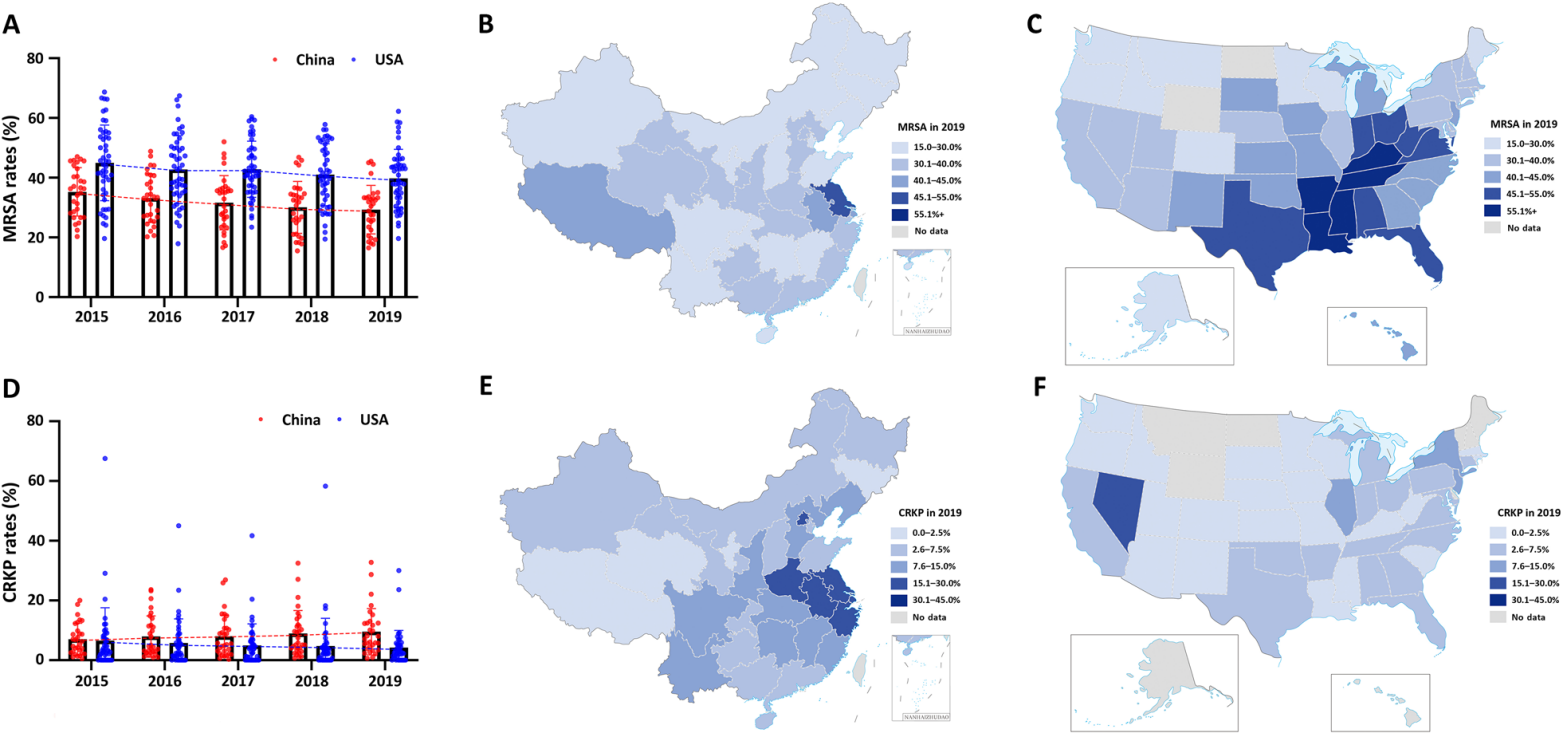
The prevalent rates of CRKP and MRSA in China and the USA during 2015‒2019. **A** MRSA positive rates (%) from 2015 to 2019 in China and USA; **B** MRSA in 2019 in China; **C** MRSA in 2019 in the USA; **D** CRKP positive rates (%) from 2015 to 2019 in China and USA; **E** CRKP in 2019 in China; **F** CRKP in 2019 in the USA; *CRKP* carbapenem-resistant *Klebsiella pneumoniae,* *MRSA* methicillin-resistant *Staphylococcus aureus*

China and the USA are the world's largest developing and developed countries with a massive population, vast geographical territory, and enormous AMR data throughout all 31 provincial-level administrative divisions (PLADs) in China or 55 states in the USA. Here, the differences in AMR prevalence across different Chinese PLADs or states in the USA are substantially more dramatic than within other countries. Thus, from 2015 to 2019, we studied AMR rates for gram-positive MRSA and gram-negative CRKP in every PLAD of China or state of the USA (Fig. 8A–F).

As shown in Fig. 8B, the prevalence of MRSA in China varies greatly throughout the country, ranging from 16.5% to 45.5%, with an average of 30.2%. Jiangsu (45.5%) and Shanxi (16.5%) have the highest and lowest prevalence, respectively. MRSA prevalence in the USA range from 19.6% to 62.2%, with an average of 40.6%. Kentucky (62.2%) and Montana (19.6%) have the highest and lowest prevalent rates, respectively. MRSA is surprisingly more prevalent in the USA than in China (Fig. 8A). Between 2015 and 2019, MRSA prevalence decreased gradually in both countries, possibly due to relatively comprehensive monitoring systems, advanced experimental techniques, and increased public education attention on the indicators AMU (ranking: 35th/146 in China and 10th/146 in the USA), TLV (14th in China and 29th in the USA), and PHA (9th in China and 10th in the USA). Additionally, as shown in Fig. 8E, F, the prevalence of CRKP varied between 0.6% and 32.8% in various PLADs of China in 2019, with an average of 10.9%. The greatest and lowest incidence rates were found in Henan (32.8%) and Tibet (0.6%), respectively. In these states of the USA, the prevalence of CRKP ranges between 0 and 30%, with an average of 4.7% and 30.0% in Puerto Rico. In contrast to a gradual reduction in the USA, CRKP prevalence increased significantly in China from 2015 to 2019 (Fig. 8D), consistent with the higher sub-score of the carbapenem-related sub-indicator CAR in the USA (87.62) than in China (56.00).

## Discussion

Consistent with the positive correlation between GOHI-AMR and GDP, GNI, and domestic health expenditures, the HICs significantly outperformed the other three income nation groups, with all five key indicators dramatically above the global average. Particularly for the key indicator ACO, nearly all of the HICs have accounted for the proper use of antibiotics not just in humans but in animals and environments as well. Simultaneously, HICs have numerous existing challenges. Firstly, unexpectedly, in the sub-indicators MR-SA and AR-ABA, the prevalence of MRSA and aminoglycosides-resistant *Acinetobacter baumannii* in HICs are significantly higher than those in LICs but lower than in UMICs and LMICs. Moreover, utilizing AMR surveillance data from ECDC, there are still some HICs, such as Cyprus and Malta, with a higher prevalence of ESKAPE and no effective preventative or control measures, demonstrating that even within HICs, huge AMR discrepancies also exist; Secondly, among the monitoring-related key indicators ASS, less than one-third of HICs scored over 60 on the indicator AMC (antibacterial drug consumption monitoring for humans and animals). The primary issue is the inadequacy of national monitoring of animal antibiotic consumption, with only New Zealand scoring 75 on the sub-indicator ACA. In the future, UMICs, LMICs, and LICs will confront similar AMR challenges.

Except for the key indicator ARR in LICs, the average scores of the remaining 4 key indicators within the other three income groups are all lower than the global average. Due to some advances in the optimization of antibiotics, particularly in animals, the key indicators ASS and ACO are marginally better in UMICs compared to LMICs and LICs. However, there are huge gaps in the monitoring of animals and plants, especially in LICs. In addition, UMICs and LMICs are striving to raise awareness of AMR within their populations. The public and AMR-related professionals, such as doctors, veterinarians, and farmers, have a greater understanding of antibiotics than those in LICs. Surprisingly, the majority of countries globally performed poorly on the ARR. Nonetheless, the performances of LICs were significantly higher than the world average and also exceeded those of UMICs and LMICs. In particular, the aminoglycoside and quinolone-resistant bacteria prevalence of LICs are even close to those of HICs. The performance of the other four key indicators, however, ranks lowest for LICs. As a result, LICs have increased their monitoring of AMR in humans and animals. National plans should include monitoring of the environment within a reasonable amount of time, and multidisciplinary collaboration should be taken into account when building scientific research platforms.

Middle-income countries, encompassing UMICs and LMICs, face unique problems compared to HICs and LICs. Brazil and Argentina are the world's two largest meat suppliers [33]. They performed particularly badly on the sub-indicators ACA and PTU due to the absence of antimicrobial consumption monitoring in the animals and antimicrobial monitoring [34], which would undoubtedly lead to the spread of AMR genes or strains across global food chains [35]. Despite its most advanced economic growth, Russia, one of the BRICS countries, has serious AMR problems, with a score of 10.99 on the key indicator ARR, which is even worse than that in sub-Saharan Africa. AMI and QUI were Russia's other low-scoring indicators, scoring 3.46 and 8.6 points, respectively. Similarly, the remaining four BRICs countries had somewhat lower sub-rankings in the same ARR. India's most significant problem was the extremely high AMR prevalence among clinical pathogens. For instance, New Delhi-Metallo-β-lactamases were discovered in an Indian patient and spread widely across Pakistan, China, the United Kingdom, and other countries worldwide [37]. India particularly needs to strengthen its national monitoring systems and networks to combat significant AMR, such as those resistant to carbapenems, β-lactams, and quinolones. China's performance ranks third among UMICs. According to a recent study, P50 (antimicrobial medicines with greater than 50% resistance) resistance is rapidly increasing in pigs and chickens in middle-income countries, including China [34]. This finding indicates that China's animal resistance surveillance systems and networks, including the sub-score of 25.00 in the sub-indicators ACA (antimicrobial consumption in animals), AMF (AMR in food) (sub-score: 50.00, sub-ranking: 54th/146), and RTF (reduce transmission of AMR during food processing; 33.33, 75th/146), require substantial improvement. Even though China's current monitoring management and scientific research capabilities perform well in UMICs, a substantial gap exists between China and the top HICs. Increasingly, monitoring in these countries will be done by region, and they will deal with the AMR problem on an individual basis by studying what makes resistance in each region distinct.

Here, we confirmed multiple GOHI-AMR-related factors. However, as the majority of high-scoring countries are HICs and UMICs, and the majority of low-scoring countries are LICs and LMICs, it is unquestionable that economic factors continue to be the most important driver of AMR governance competence. Surprisingly, a few impoverished countries that implemented an early One Health-based response against AMR scored far higher on the GOHI-AMR than a great number of developed countries. Malaysia, for instance, developed a significant number of human, animal, and environmental AMR-related government departments in 1988 as part of its "National Surveillance of Antibiotic Resistance" initiative [37]. Similarly, the Thai government announced a One Health strategy to tackle AMR in 2015, including monitoring antimicrobial usage, antibiotic stewardship, and infection control, preventing the spread of AMR bacteria, and raising public awareness [38, 39]. The positive correlation with life expectancy also indicates that AMR has become an important factor affecting human life expectancy in the current environment of severe drug resistance. In this study, forest coverage was the only environmental factor that indicated no correlation with GOHI-AMR. Studies have revealed the influence of temperature and environment on AMR, such as climatic variables impacting the incidence of MRSA skin and soft tissue infections [40]. In addition, inland and offshore *E. coli* resistance patterns are distinct. We will consider more environmental aspects in future studies [41].

The country's overall rankings under GHS-AMR and GOHI-AMR are nearly the same for the vast majority of European HICs. However, several UMICs with low GOHI-AMR overall rankings, such as Brazil (overall ranking: 88th/146 in GOHI-AMR and 12th/195 in GHS-AMR), Argentina (81st/146 and 12th/195), and Armenia (107th/146 and 12th/195), scored rarely higher in GHS-AMR. Nonetheless, based on the sub-indicators ACH, ACA, RDT, and MSW originating from TrACSS, these differentiated-ranking countries have particularly severe deficiencies in animal and human antimicrobial consumption control, as well as multi-platform collaboration [36]. In addition, we developed a new nationwide indicator that depicts the actual prevalence of AMR bacteria, together with other updated indicators for humans, animals, and the environment. In the meantime, AMR monitoring data from WHO's GLASS indicated severe local epidemic patterns of multiple AMR bacteria in the aforementioned countries. Hence, these divergent country rankings between GOHI-AMR and GHS-AMR are mostly attributed to the newly developed quantitative AMR indicators on the actual prevalence of the AMR bacteria, demonstrating the unique advantages of GOHI-AMR. In addition, unlike TrACSS, GOHI-AMR quantifies questionnaire results across multiple One Health categories and gives direct comparison rankings of a country's investigation outcomes. So, based on the GOHI-AMR indicator system, each country can be given a global AMR overall rating and precise scientific measurements and recommendations, as well as the differences between itself and the best countries.

This index system relies heavily on official international or national databases, such as the WHO's GLASS, GHS, and TrACSS, as well as authoritative monitoring databases, such as CARSS in China, the USA CDC, and the European CDC. AMR datasets in these industrialized countries are quite complete, whereas the majority of LICs and LMICs lack substantial AMR data. Moreover, the current analysis adopts an indicator evaluation system that requires at least one year of data; the most recent data from GHS in 2021, TrACSS and Europe CDC in 2020 were collected. The rest of the data including GLASS is from 2019, which shows that there hasn't been a lack of continuous dynamic change throughout the evaluation, and the data utilized is from several years ago, which makes it impossible to compare horizontally.

## Conclusions

This study is, to the best of our knowledge, the most comprehensive investigation to date of global AMR status within the framework of One Health. In particular, our findings demonstrate that AMR is still a serious global health concern, especially in LICs and LMICs, such as in sub-Saharan Africa. Meanwhile, establishing laboratory infrastructure and multidisciplinary platforms rapidly will be critical to addressing the enormous burden of AMR. Simultaneously, this will urgently require more extensive surveillance of AMR in humans, animals, and the surrounding environment.

## Supplementary Information

**Additional file 1:** **Table S1**: Technical file for GOHI-AMR.

**Additional file 2:** **Table S2**: Index framework for GOHI-AMR. **Table S3:** Global rankings for 146 countries worldwide. **Table S4:** All the indicator scores for 146 countries worldwide.

## Data Availability

Not applicable.

## Abbreviations

AMR: Antimicrobial resistance
CARSS: China Antimicrobial Resistance Surveillance System
COVID-19: Coronavirus Disease 2019
ECDC: European Centre for Disease Prevention and Control
FAHP: Fuzzy Analytical Hierarchy Process
FAO: Food and Agriculture Organization
GHS: Global health security index
GLASS: Global Antimicrobial Resistance and Use Surveillance System
GOHI: Global One Health Index
HICs: High-income countries
LICs: Low-income countries
LMICs: Lower-middle-income countries
OIE: World Organization for Animal Health
TrACSS: Tripartite AMR country self-assessment survey
UMICs: Upper-middle-income countries
WHO: World Health Organization
