# Correction: Global antimicrobial resistance: a system-wide comprehensive investigation using the Global One Health Index

**DOI:** 10.1186/s40249-022-01027-2

**Published:** 2022-09-22

**Authors:** Nan Zhou, Zile Cheng, Xiaoxi Zhang, Chao Lv, Chaoyi Guo, Haodong Liu, Ke Dong, Yan Zhang, Chang Liu, Yung-Fu Chang, Sheng Chen, Xiaokui Guo, Xiao-Nong Zhou, Min Li, Yongzhang Zhu

**Affiliations:** 1 Department of Animal Health and Food Safety, School of Global Health, Chinese Center for Tropical Diseases Research, Shanghai Jiao Tong University School of Medicine, Shanghai, 200025 China; 2 One Health Center, Shanghai Jiao Tong University-The University of Edinburgh, Shanghai, 200025 China; 3 National Institute of Parasitic Diseases at Chinese Center for Disease Control and Prevention (Chinese Center for Tropical Diseases Research), NHC Key Laboratory of Parasite and Vector Biology, WHO Collaborating Centre for Tropical Diseases, Shanghai, China; 4 Department of Population Medicine and Diagnostic Sciences, College of Veterinary Medicine, Cornell University, Ithaca, NY USA; 5 Department of Infectious Diseases and Public Health, Jockey Club College of Veterinary Medicine and Life Sciences, City University of Hong Kong, Kowloon, Hong Kong China

## Correction: Infectious Diseases of Poverty (2022) 11:92 10.1186/s40249-022-01016-5

Correction

After publication of this article [1], it was brought to our attention that one of the authors’ name was incorrectly spelt. The correct name of the author should be Yung-Fu Chang, rather than YunFu Chang.

The original publication has been corrected.
