# Supplementary material for: Global antimicrobial resistance: a system-wide comprehensive investigation using the Global One Health Index
Source: Infect Dis Poverty. 2022 Aug 23;11:92. doi: 10.1186/s40249-022-01016-5 (PMC9395850; doi:10.1186/s40249-022-01016-5)
Supplement: Supplementary file 1 — Additional file 1: Table S1: Technical file for GOHI-AMR. [file 40249_2022_1016_MOESM1_ESM.docx]

Technical File for GOHI-AMR

| Indicator Title | Antimicrobial consumption in human |
| --- | --- |
| Indicator code | 1.1.1 |
| Raw Data Title | National monitoring system for consumption of antimicrobials in human health |
| Raw Data Abbreviation | ACH |
| Value Range | (0, 4) |
| Years Available | 2020 |
| Countries Available | 135 |
| Database | Global Database for the Tripartite Antimicrobial Resistance (AMR) Country Self-assessment Survey (TrACSS) |
| Published by | WHO, OIE, FAO |
| Download Link | <https://amrcountryprogress.org/#/download-responses> |
| Data Citation | WHO, OIE, FAO, Global Database for the Tripartite Antimicrobial Resistance (AMR) Country Self-assessment Survey (TrACSS) [Data file].Retrieved from: <https://amrcountryprogress.org/#/download-responses> |

| Indicator Title | Antimicrobial consumption in animals |
| --- | --- |
| Indicator code | 1.1.2 |
| Raw Data Title | National monitoring system for consumption of antimicrobials in animals (terrestrial and aquatic) |
| Raw Data Abbreviation | ACA |
| Value Range | (0, 4) |
| Years Available | 2020 |
| Countries Available | 131 |
| Database | Global Database for the Tripartite Antimicrobial Resistance (AMR) Country Self-assessment Survey (TrACSS) |
| Published by | WHO, OIE, FAO |
| Download Link | <https://amrcountryprogress.org/#/download-responses> |
| Data Citation | WHO, OIE, FAO, Global Database for the Tripartite Antimicrobial Resistance (AMR) Country Self-assessment Survey (TrACSS) [Data file].Retrieved from: <https://amrcountryprogress.org/#/download-responses> |

| Indicator Title | Pesticide Use |
| --- | --- |
| Indicator code | 1.1.3 |
| Raw Data Title | National monitoring system for consumption of antimicrobials in animals (terrestrial and aquatic) |
| Raw Data Abbreviation | PTU |
| Value Range | (0, 4) |
| Years Available | 2020 |
| Countries Available | 124 |
| Database | Global Database for the Tripartite Antimicrobial Resistance (AMR) Country Self-assessment Survey (TrACSS) |
| Published by | WHO, OIE, FAO |
| Download Link | <https://amrcountryprogress.org/#/download-responses> |
| Data Citation | WHO, OIE, FAO, Global Database for the Tripartite Antimicrobial Resistance (AMR) Country Self-assessment Survey (TrACSS) [Data file].Retrieved from: <https://amrcountryprogress.org/#/download-responses> |

| Indicator Title | AMR in human |
| --- | --- |
| Indicator Affiliation | AMH/AMU/ ASS/AMR/CDI |
| Indicator code | 1.2.1 |
| Raw Data Title | National surveillance system for antimicrobial resistance (AMR) in humans |
| Raw Data Abbreviation | AMH |
| Value Range | (0, 4) |
| Years Available | 2020 |
| Countries Available | 134 |
| Database | Global Database for the Tripartite Antimicrobial Resistance (AMR) Country Self-assessment Survey (TrACSS) |
| Published by | WHO, OIE, FAO |
| Download Link | <https://amrcountryprogress.org/#/download-responses> |
| Data Citation | WHO, OIE, FAO, Global Database for the Tripartite Antimicrobial Resistance (AMR) Country Self-assessment Survey (TrACSS) [Data file].Retrieved from: <https://amrcountryprogress.org/#/download-responses> |

| Indicator Title | AMR in animals |
| --- | --- |
| Indicator code | 1.2.2 |
| Raw Data Title | National surveillance system for antimicrobial resistance (AMR) in animals (terrestrial and aquatic) |
| Raw Data Abbreviation | AMA |
| Value Range | (0, 4) |
| Years Available | 2020 |
| Countries Available | 132 |
| Database | Global Database for the Tripartite Antimicrobial Resistance (AMR) Country Self-assessment Survey (TrACSS) |
| Published by | WHO, OIE, FAO |
| Download Link | <https://amrcountryprogress.org/#/download-responses> |
| Data Citation | WHO, OIE, FAO, Global Database for the Tripartite Antimicrobial Resistance (AMR) Country Self-assessment Survey (TrACSS) [Data file].Retrieved from: <https://amrcountryprogress.org/#/download-responses> |

| Indicator Title | AMR in food |
| --- | --- |
| Indicator code | 1.2.3 |
| Raw Data Title | National surveillance system for antimicrobial resistance (AMR) in food (animal and plant origin) |
| Raw Data Abbreviation | AMF |
| Value Range | (0, 4) |
| Years Available | 2020 |
| Countries Available | 134 |
| Database | Global Database for the Tripartite Antimicrobial Resistance (AMR) Country Self-assessment Survey (TrACSS) |
| Published by | WHO, OIE, FAO |
| Download Link | <https://amrcountryprogress.org/#/download-responses> |
| Data Citation | WHO, OIE, FAO, Global Database for the Tripartite Antimicrobial Resistance (AMR) Country Self-assessment Survey (TrACSS) [Data file].Retrieved from: <https://amrcountryprogress.org/#/download-responses> |

| Indicator Title | Environmental surveillance system |
| --- | --- |
| Indicator code | 1.3.1 |
| Raw Data Title | National environmental surveillance system for antimicrobial residues in soil and waterways |
| Raw Data Abbreviation | ESS |
| Value Range | (0, 1) |
| Years Available | 2021 |
| Countries Available | 194 |
| Database | Global Health Security Index |
| Published by | Nuclear Threat Initiative, Center for Health Security, Johns Hopkins Bloomberg School of Public Health |
| Download Link | <https://www.ghsindex.org/> |
| Data Citation | Nuclear Threat Initiative, Center for Health Security, Johns Hopkins Bloomberg School of Public Health. Global Health Security Index (2019) [Data file]. Retrieved from: <https://www.ghsindex.org/> |

| Indicator Title | National surveillance sites |
| --- | --- |
| Indicator code | 2.1.1 |
| Raw Data Title | Establishment of national reference laboratory |
| Raw Data Abbreviation | NRL |
| Value Range | (0.171, 0.818) |
| Years Available | 2020 |
| Countries Available | 6 regions |
| Database | Global Antimicrobial Resistance and Use Surveillance System (GLASS) |
|  |  |
| Published by | WHO |
| Download Link | <https://www.who.int/data/gho/data/themes/topics/global-antimicrobial-resistance-surveillance-system-glass> |
| Data Citation | WHO. Global Antimicrobial Resistance and Use Surveillance System (GLASS). Retrieved from: <https://www.who.int/data/gho/data/themes/topics/global-antimicrobial-resistance-surveillance-system-glass> |

| Indicator Title | Effective integration of laboratories |
| --- | --- |
| Indicator Affiliation | EIL/TLV/LNC/AMR/CDI |
| Indicator code | 2.1.2 |
| Raw Data Title | Effective integration of laboratories in the AMR surveillanc |
| Raw Data Abbreviation | EIL |
| Value Range | (0, 4) |
| Years Available | 2020 |
| Countries Available | 134 |
| Database | Global Database for the Tripartite Antimicrobial Resistance (AMR) Country Self-assessment Survey (TrACSS) |
| Published by | WHO, OIE, FAO |
| Download Link | <https://amrcountryprogress.org/#/download-responses> |
| Data Citation | WHO, OIE, FAO, Global Database for the Tripartite Antimicrobial Resistance (AMR) Country Self-assessment Survey (TrACSS) [Data file].Retrieved from: <https://amrcountryprogress.org/#/download-responses> |

| Indicator Title | Multi-sector working |
| --- | --- |
| Indicator code | 2.2.1 |
| Raw Data Title | Multi-sector and One Health collaboration/coordination |
| Raw Data Abbreviation | MSW |
| Value Range | (0, 4) |
| Years Available | 2020 |
| Countries Available | 133 |
| Database | Global Database for the Tripartite Antimicrobial Resistance (AMR) Country Self-assessment Survey (TrACSS) |
| Published by | WHO, OIE, FAO |
| Download Link | <https://amrcountryprogress.org/#/download-responses> |
| Data Citation | WHO, OIE, FAO, Global Database for the Tripartite Antimicrobial Resistance (AMR) Country Self-assessment Survey (TrACSS) [Data file].Retrieved from: <https://amrcountryprogress.org/#/download-responses> |

| Indicator Title | Standardization and harmonization of laboratories |
| --- | --- |
| Indicator code | 2.2.2 |
| Raw Data Title | Level of the standardization and harmonization of procedures among laboratories |
| Raw Data Abbreviation | SHL |
| Value Range | (0, 4) |
| Years Available | 2020 |
| Countries Available | 133 |
| Database | Global Database for the Tripartite Antimicrobial Resistance (AMR) Country Self-assessment Survey (TrACSS) |
| Published by | WHO, OIE, FAO |
| Download Link | <https://amrcountryprogress.org/#/download-responses> |
| Data Citation | WHO, OIE, FAO, Global Database for the Tripartite Antimicrobial Resistance (AMR) Country Self-assessment Survey (TrACSS) [Data file].Retrieved from: <https://amrcountryprogress.org/#/download-responses> |

| Indicator Title | Relevance of diagnostic techniques |
| --- | --- |
| Indicator Affiliation | RDT/TLV/LNC/AMR/CDI |
| Indicator code | 2.2.3 |
| Raw Data Title | Relevance of diagnostic (bacteriology) techniques used by laboratories included in the AMR surveillance system |
| Raw Data Abbreviation | RDT |
| Value Range | (0, 4) |
| Years Available | 2020 |
| Countries Available | 132 |
| Database | Global Database for the Tripartite Antimicrobial Resistance (AMR) Country Self-assessment Survey (TrACSS) |
| Published by | WHO, OIE, FAO |
| Download Link | <https://amrcountryprogress.org/#/download-responses> |
| Data Citation | WHO, OIE, FAO, Global Database for the Tripartite Antimicrobial Resistance (AMR) Country Self-assessment Survey (TrACSS) [Data file].Retrieved from: <https://amrcountryprogress.org/#/download-responses> |

| Indicator Title | Technical level of data management |
| --- | --- |
| Indicator Affiliation | LDM/TLV/LNC/AMR/CDI |
| Indicator code | 2.2.4 |
| Raw Data Title | Technical level of data management of the laboratory network in the AMR surveillance system |
| Raw Data Abbreviation | LDM |
| Value Range | (0, 4) |
| Years Available | 2020 |
| Countries Available | 132 |
| Database | Global Database for the Tripartite Antimicrobial Resistance (AMR) Country Self-assessment Survey (TrACSS) |
| Published by | WHO, OIE, FAO |
| Download Link | <https://amrcountryprogress.org/#/download-responses> |
| Data Citation | WHO, OIE, FAO, Global Database for the Tripartite Antimicrobial Resistance (AMR) Country Self-assessment Survey (TrACSS) [Data file].Retrieved from: <https://amrcountryprogress.org/#/download-responses> |

| Indicator Title | National plan for AMR priority pathogens |
| --- | --- |
| Indicator code | 2.3.1 |
| Raw Data Title | Is there a national AMR plan for the surveillance, detection and reporting of priority AMR pathogens |
| Raw Data Abbreviation | NPP |
| Value Range | (0, 2) |
| Years Available | 2019,2021 |
| Countries Available | 194 |
| Database | Global Health Security Index |
| Published by | Nuclear Threat Initiative, Center for Health Security, Johns Hopkins Bloomberg School of Public Health |
| Download Link | <https://www.ghsindex.org/> |
| Data Citation | Nuclear Threat Initiative, Center for Health Security, Johns Hopkins Bloomberg School of Public Health. Global Health Security Index (2019) [Data file]. Retrieved from: <https://www.ghsindex.org/> |

| Indicator Title | National action plan on AMR |
| --- | --- |
| Indicator code | 2.3.2 |
| Raw Data Title | Country progress with development of a national action plan on AMR |
| Raw Data Abbreviation | NAP |
| Value Range | (0, 4) |
| Years Available | 2020 |
| Countries Available | 136 |
| Database | Global Database for the Tripartite Antimicrobial Resistance (AMR) Country Self-assessment Survey (TrACSS) |
| Published by | WHO, OIE, FAO |
| Download Link | <https://amrcountryprogress.org/#/download-responses> |
| Data Citation | WHO, OIE, FAO, Global Database for the Tripartite Antimicrobial Resistance (AMR) Country Self-assessment Survey (TrACSS) [Data file].Retrieved from: <https://amrcountryprogress.org/#/download-responses> |

| Indicator Title | National action plan on AMR linked to any other existing action plans |
| --- | --- |
| Indicator code | 2.3.3 |
| Raw Data Title | Whether national action plan on AMR linked to any other existing action plans |
| Raw Data Abbreviation | LOE |
| Value Range | (0, 4) |
| Years Available | 2020 |
| Countries Available | 136 |
| Database | Global Database for the Tripartite Antimicrobial Resistance (AMR) Country Self-assessment Survey (TrACSS) |
| Published by | WHO, OIE, FAO |
| Download Link | <https://amrcountryprogress.org/#/download-responses> |
| Data Citation | WHO, OIE, FAO, Global Database for the Tripartite Antimicrobial Resistance (AMR) Country Self-assessment Survey (TrACSS) [Data file].Retrieved from: <https://amrcountryprogress.org/#/download-responses> |

| Indicator Title | Publishment of action plan |
| --- | --- |
| Indicator code | 2.3.4 |
| Raw Data Title | Whether published their own national action plan on AMR |
| Raw Data Abbreviation | PAP |
| Value Range | (0, 4) |
| Years Available | 2020 |
| Countries Available | 80 |
| Database | Global Database for the Tripartite Antimicrobial Resistance (AMR) Country Self-assessment Survey (TrACSS) |
| Published by | WHO, OIE, FAO |
| Download Link | <https://amrcountryprogress.org/#/download-responses> |
| Data Citation | WHO, OIE, FAO, Global Database for the Tripartite Antimicrobial Resistance (AMR) Country Self-assessment Survey (TrACSS) [Data file].Retrieved from: <https://amrcountryprogress.org/#/download-responses> |

| Indicator Title | National law(s) for antibiotic use in humans |
| --- | --- |
| Indicator Affiliation | LUH/NLA/ACO/AMR/CDI |
| Indicator code | 3.1.1 |
| Raw Data Title | Is there national legislation or regulation in place requiring prescriptions for antibiotic use for humans |
| Raw Data Abbreviation | LUH |
| Value Range | (0, 1) |
| Years Available | 2021 |
| Countries Available | 194 |
| Database | Global Health Security Index |
| Published by | Nuclear Threat Initiative, Center for Health Security, Johns Hopkins Bloomberg School of Public Health |
| Download Link | <https://www.ghsindex.org/> |
| Data Citation | Nuclear Threat Initiative, Center for Health Security, Johns Hopkins Bloomberg School of Public Health. Global Health Security Index (2019) [Data file]. Retrieved from: <https://www.ghsindex.org/> |

| Indicator Title | National law(s) for antibiotic use in animals |
| --- | --- |
| Indicator code | 3.1.2 |
| Raw Data Title | Is there national legislation or regulation in place requiring prescriptions for antibiotic use for animals |
| Raw Data Abbreviation | LUA |
| Value Range | (0, 1) |
| Years Available | 2021 |
| Countries Available | 194 |
| Database | Global Health Security Index |
| Published by | Nuclear Threat Initiative, Center for Health Security, Johns Hopkins Bloomberg School of Public Health |
| Download Link | <https://www.ghsindex.org/> |
| Data Citation | Nuclear Threat Initiative, Center for Health Security, Johns Hopkins Bloomberg School of Public Health. Global Health Security Index (2019) [Data file]. Retrieved from: <https://www.ghsindex.org/> |

| Indicator Title | National law(s) on marketing of pesticides |
| --- | --- |
| Indicator code | 3.1.3 |
| Raw Data Title | Whether country has legislation on marketing of pesticides including antimicrobial pesticides, such as bactericides and fungicides used in plant production |
| Raw Data Abbreviation | NLM |
| Value Range | (0, 1) |
| Years Available | 2020 |
| Countries Available | 136 |
| Database | Global Database for the Tripartite Antimicrobial Resistance (AMR) Country Self-assessment Survey (TrACSS) |
| Published by | WHO, OIE, FAO |
| Download Link | <https://amrcountryprogress.org/#/download-responses> |
| Data Citation | WHO, OIE, FAO, Global Database for the Tripartite Antimicrobial Resistance (AMR) Country Self-assessment Survey (TrACSS) [Data file].Retrieved from: <https://amrcountryprogress.org/#/download-responses> |

| Indicator Title | National law(s) on prohibits the use of antibiotics |
| --- | --- |
| Indicator code | 3.1.4 |
| Raw Data Title | Whether country has laws or regulations that prohibits the use of antibiotics for growth promotion in the absence of risk analysis |
| Raw Data Abbreviation | NLP |
| Value Range | (0, 1) |
| Years Available | 2020 |
| Countries Available | 136 |
| Database | Global Database for the Tripartite Antimicrobial Resistance (AMR) Country Self-assessment Survey (TrACSS) |
| Published by | WHO, OIE, FAO |
| Download Link | <https://amrcountryprogress.org/#/download-responses> |
| Data Citation | WHO, OIE, FAO, Global Database for the Tripartite Antimicrobial Resistance (AMR) Country Self-assessment Survey (TrACSS) [Data file].Retrieved from: <https://amrcountryprogress.org/#/download-responses> |

| Indicator Title | Optimizing antimicrobial use in human health |
| --- | --- |
| Indicator code | 3.2.1 |
| Raw Data Title | Optimizing antimicrobial use in human health |
| Raw Data Abbreviation | OUH |
| Value Range | (0, 4) |
| Years Available | 2020 |
| Countries Available | 135 |
| Database | Global Database for the Tripartite Antimicrobial Resistance (AMR) Country Self-assessment Survey (TrACSS) |
| Published by | WHO, OIE, FAO |
| Download Link | <https://amrcountryprogress.org/#/download-responses> |
| Data Citation | WHO, OIE, FAO, Global Database for the Tripartite Antimicrobial Resistance (AMR) Country Self-assessment Survey (TrACSS) [Data file].Retrieved from: <https://amrcountryprogress.org/#/download-responses> |

| Indicator Title | Optimizing antimicrobial use in animal health |
| --- | --- |
| Indicator code | 3.2.2 |
| Raw Data Title | Optimizing antimicrobial use in animal health (terrestrial and aquatic) |
| Raw Data Abbreviation | OUA |
| Value Range | (0, 4) |
| Years Available | 2020 |
| Countries Available | 133 |
| Database | Global Database for the Tripartite Antimicrobial Resistance (AMR) Country Self-assessment Survey (TrACSS) |
| Published by | WHO, OIE, FAO |
| Download Link | <https://amrcountryprogress.org/#/download-responses> |
| Data Citation | WHO, OIE, FAO, Global Database for the Tripartite Antimicrobial Resistance (AMR) Country Self-assessment Survey (TrACSS) [Data file].Retrieved from: <https://amrcountryprogress.org/#/download-responses> |

| Indicator Title | Optimizing antimicrobial pesticide use in plant |
| --- | --- |
| Indicator code | 3.2.3 |
| Raw Data Title | Optimizing antimicrobial pesticide use in plant production |
| Raw Data Abbreviation | OAP |
| Value Range | (0, 4) |
| Years Available | 2020 |
| Countries Available | 123 |
| Database | Global Database for the Tripartite Antimicrobial Resistance (AMR) Country Self-assessment Survey (TrACSS) |
| Published by | WHO, OIE, FAO |
| Download Link | <https://amrcountryprogress.org/#/download-responses> |
| Data Citation | WHO, OIE, FAO, Global Database for the Tripartite Antimicrobial Resistance (AMR) Country Self-assessment Survey (TrACSS) [Data file].Retrieved from: <https://amrcountryprogress.org/#/download-responses> |

| Indicator Title | Infection Prevention and Control in human |
| --- | --- |
| Indicator code | 3.3.1 |
| Raw Data Title | Infection Prevention and Control in human health care |
| Raw Data Abbreviation | IPC |
| Value Range | (0, 4) |
| Years Available | 2020 |
| Countries Available | 135 |
| Database | Global Database for the Tripartite Antimicrobial Resistance (AMR) Country Self-assessment Survey (TrACSS) |
| Published by | WHO, OIE, FAO |
| Download Link | <https://amrcountryprogress.org/#/download-responses> |
| Data Citation | WHO, OIE, FAO, Global Database for the Tripartite Antimicrobial Resistance (AMR) Country Self-assessment Survey (TrACSS) [Data file].Retrieved from: <https://amrcountryprogress.org/#/download-responses> |

| Indicator Title | Reduce transmission of AMR in animal production |
| --- | --- |
| Indicator code | 3.3.2 |
| Raw Data Title | Good health, management and hygiene practices to reduce the use of antimicrobials and minimize development and transmission of AMR in animal production (terrestrial and aquatic) |
| Raw Data Abbreviation | RTA |
| Value Range | (0, 4) |
| Years Available | 2020 |
| Countries Available | 133 |
| Database | Global Database for the Tripartite Antimicrobial Resistance (AMR) Country Self-assessment Survey (TrACSS) |
| Published by | WHO, OIE, FAO |
| Download Link | <https://amrcountryprogress.org/#/download-responses> |
| Data Citation | WHO, OIE, FAO, Global Database for the Tripartite Antimicrobial Resistance (AMR) Country Self-assessment Survey (TrACSS) [Data file].Retrieved from: <https://amrcountryprogress.org/#/download-responses> |

| Indicator Title | Reduce transmission of AMR in food processing |
| --- | --- |
| Indicator code | 3.3.3 |
| Raw Data Title | Good management and hygiene practices to reduce the development and transmission of AMR in food processing |
| Raw Data Abbreviation | RTF |
| Value Range | (0, 4) |
| Years Available | 2020 |
| Countries Available | 133 |
| Database | Global Database for the Tripartite Antimicrobial Resistance (AMR) Country Self-assessment Survey (TrACSS) |
| Published by | WHO, OIE, FAO |
| Download Link | <https://amrcountryprogress.org/#/download-responses> |
| Data Citation | WHO, OIE, FAO, Global Database for the Tripartite Antimicrobial Resistance (AMR) Country Self-assessment Survey (TrACSS) [Data file].Retrieved from: <https://amrcountryprogress.org/#/download-responses> |

| Indicator Title | Raising awareness and understanding |
| --- | --- |
| Indicator code | 4.1.1 |
| Raw Data Title | Raising awareness and understanding of AMR |
| Raw Data Abbreviation | RAU |
| Value Range | (0, 4) |
| Years Available | 2020 |
| Countries Available | 136 |
| Database | Global Database for the Tripartite Antimicrobial Resistance (AMR) Country Self-assessment Survey (TrACSS) |
| Published by | WHO, OIE, FAO |
| Download Link | <https://amrcountryprogress.org/#/download-responses> |
| Data Citation | WHO, OIE, FAO, Global Database for the Tripartite Antimicrobial Resistance (AMR) Country Self-assessment Survey (TrACSS) [Data file].Retrieved from: <https://amrcountryprogress.org/#/download-responses> |

| Indicator Title | Training in the human health sector |
| --- | --- |
| Indicator code | 4.2.1 |
| Raw Data Title | Training and professional education on AMR in the human health sector |
| Raw Data Abbreviation | THS |
| Value Range | (0, 4) |
| Years Available | 2020 |
| Countries Available | 135 |
| Database | Global Database for the Tripartite Antimicrobial Resistance (AMR) Country Self-assessment Survey (TrACSS) |
| Published by | WHO, OIE, FAO |
| Download Link | <https://amrcountryprogress.org/#/download-responses> |
| Data Citation | WHO, OIE, FAO, Global Database for the Tripartite Antimicrobial Resistance (AMR) Country Self-assessment Survey (TrACSS) [Data file].Retrieved from: <https://amrcountryprogress.org/#/download-responses> |

| Indicator Title | Training in the veterinary sector |
| --- | --- |
| Indicator code | 4.2.2 |
| Raw Data Title | Training and professional education on AMR in the human health sector |
| Raw Data Abbreviation | TVS |
| Value Range | (0, 4) |
| Years Available | 2020 |
| Countries Available | 133 |
| Database | Global Database for the Tripartite Antimicrobial Resistance (AMR) Country Self-assessment Survey (TrACSS) |
| Published by | WHO, OIE, FAO |
| Download Link | <https://amrcountryprogress.org/#/download-responses> |
| Data Citation | WHO, OIE, FAO, Global Database for the Tripartite Antimicrobial Resistance (AMR) Country Self-assessment Survey (TrACSS) [Data file].Retrieved from: <https://amrcountryprogress.org/#/download-responses> |

| Indicator Title | Training in farming sector |
| --- | --- |
| Indicator code | F.4.2.3 |
| Raw Data Title | Training and professional education on AMR in farming sector (animal and plant), food production, food safety and the environmen |
| Raw Data Abbreviation | TFS |
| Value Range | (0, 4) |
| Years Available | 2020 |
| Countries Available | 132 |
| Database | Global Database for the Tripartite Antimicrobial Resistance (AMR) Country Self-assessment Survey (TrACSS) |
| Published by | WHO, OIE, FAO |
| Download Link | <https://amrcountryprogress.org/#/download-responses> |
| Data Citation | WHO, OIE, FAO, Global Database for the Tripartite Antimicrobial Resistance (AMR) Country Self-assessment Survey (TrACSS) [Data file].Retrieved from: <https://amrcountryprogress.org/#/download-responses> |

| Indicator Title | Progress with strengthening veterinary services |
| --- | --- |
| Indicator code | F.4.2.4 |
| Raw Data Title | Progress with strengthening veterinary services |
| Raw Data Abbreviation | PSV |
| Value Range | (0, 4) |
| Years Available | 2020 |
| Countries Available | 133 |
| Database | Global Database for the Tripartite Antimicrobial Resistance (AMR) Country Self-assessment Survey (TrACSS) |
| Published by | WHO, OIE, FAO |
| Download Link | <https://amrcountryprogress.org/#/download-responses> |
| Data Citation | WHO, OIE, FAO, Global Database for the Tripartite Antimicrobial Resistance (AMR) Country Self-assessment Survey (TrACSS) [Data file].Retrieved from: <https://amrcountryprogress.org/#/download-responses> |

| Indicator Title | Carbapenems-resistent *Klebsiella pneumoniae* |
| --- | --- |
| Indicator code | 5.1.1 |
| Raw Data Title | Carbapenems-resistent Klebsiella pneumoniae |
| Raw Data Abbreviation | CR-KPN |
| Value Range | (0, 67.85%) |
| Years Available | 2020 |
| Countries Available | 90 |
| Data Calculation/score Rule | In order of the resistance rate from low to high, the top 10% score is set as 100, and the bottom 10% score is set as 0 |
| Data Source | National antimicrobial resistance (AMR) surveillance systems coordination capacity (who.int); PAHO/WHO Data - 3. Resistance percentages for selected pathogens; Surveillance Atlas of Infectious Diseases (europa.eu); Australian group on antimicrobial resistance - AGAR Reports; China Antimicrobial Resistance Surveillance System (<http://www.carss.cn/sys/Htmls/dist/index.html#/>) ; |

| Indicator Title | Carbapenems-resistent *Acinetobacter baumannii* |
| --- | --- |
| Indicator code | 5.1.2 |
| Raw Data Title | Carbapenems-resistent *Acinetobacter baumannii* |
| Raw Data Abbreviation | CR-ABA |
| Value Range | (0, 97.80%) |
| Years Available | 2020 |
| Countries Available | 82 |
| Data Calculation/score Rule | In order of the resistance rate from low to high, the top 10% score is set as 100, and the bottom 10% score is set as 0 |
| Data Source | National antimicrobial resistance (AMR) surveillance systems coordination capacity (who.int); PAHO/WHO Data - 3. Resistance percentages for selected pathogens; Surveillance Atlas of Infectious Diseases (europa.eu); Australian group on antimicrobial resistance - AGAR Reports; China Antimicrobial Resistance Surveillance System (<http://www.carss.cn/sys/Htmls/dist/index.html#/>) ; |

| Indicator Title | Carbapenems-resistent *Escherichia coli* |
| --- | --- |
| Indicator code | 5.1.3 |
| Raw Data Title | Carbapenems-resistent *Escherichia coli* |
| Raw Data Abbreviation | CR-ECO |
| Value Range | (0, 81.50%) |
| Years Available | 2020 |
| Countries Available | 77 |
| Data Calculation/score Rule | In order of the resistance rate from low to high, the top 10% score is set as 100, and the bottom 10% score is set as 0 |
| Data Source | National antimicrobial resistance (AMR) surveillance systems coordination capacity (who.int); PAHO/WHO Data - 3. Resistance percentages for selected pathogens; Surveillance Atlas of Infectious Diseases (europa.eu); Australian group on antimicrobial resistance - AGAR Reports; China Antimicrobial Resistance Surveillance System (<http://www.carss.cn/sys/Htmls/dist/index.html#/>) ; |

| Indicator Title | Carbapenems-resistent *Pseudomonas aeruginosa* |
| --- | --- |
| Indicator code | 5.1.4 |
| Raw Data Title | Carbapenems-resistent *Pseudomonas aeruginosa* |
| Raw Data Abbreviation | CR-PAE |
| Value Range | (3.60%, 77.20%) |
| Years Available | 2020 |
| Countries Available | 43 |
| Data Calculation/score Rule | In order of the resistance rate from low to high, the top 10% score is set as 100, and the bottom 10% score is set as 0 |
| Data Source | PAHO/WHO Data - 3. Resistance percentages for selected pathogens; Surveillance Atlas of Infectious Diseases (europa.eu); Australian group on antimicrobial resistance - AGAR Reports; China Antimicrobial Resistance Surveillance System (<http://www.carss.cn/sys/Htmls/dist/index.html#/>) ; |

| Indicator Title | Vancomycin-resistent *Enterococcus faecium* |
| --- | --- |
| Indicator code | 5.2.1 |
| Raw Data Title | Vancomycin-resistent *Enterococcus faecium* |
| Raw Data Abbreviation | VR-EFM |
| Value Range | (0, 56.60%) |
| Years Available | 2020 |
| Countries Available | 31 |
| Data Calculation/score Rule | In order of the resistance rate from low to high, the top 10% score is set as 100, and the bottom 10% score is set as 0 |
| Data Source | PAHO/WHO Data - 3. Resistance percentages for selected pathogens; Surveillance Atlas of Infectious Diseases (europa.eu); Australian group on antimicrobial resistance - AGAR Reports; China Antimicrobial Resistance Surveillance System (<http://www.carss.cn/sys/Htmls/dist/index.html#/>) ; |

| Indicator Title | Vancomycin-resistent *Enterococcus faecalis* |
| --- | --- |
| Indicator code | 5.2.2 |
| Raw Data Title | Vancomycin-resistent *Enterococcus faecalis* |
| Raw Data Abbreviation | VR-EFC |
| Value Range | (0, 7.20%) |
| Years Available | 2020 |
| Countries Available | 31 |
| Data Calculation/score Rule | In order of the resistance rate from low to high, the top 10% score is set as 100, and the bottom 10% score is set as 0 |
| Data Source | Surveillance Atlas of Infectious Diseases (europa.eu); Australian group on antimicrobial resistance - AGAR Reports; China Antimicrobial Resistance Surveillance System (<http://www.carss.cn/sys/Htmls/dist/index.html#/>) ; |

| Indicator Title | methicillin-resistant *Staphylococcus aureus* |
| --- | --- |
| Indicator code | 5.3.1 |
| Raw Data Title | Methicillin-resistant *Staphylococcus aureus* |
| Raw Data Abbreviation | MR-SA |
| Value Range | (0, 100.00%) |
| Years Available | 2020 |
| Countries Available | 70 |
| Data Calculation/score Rule | In order of the resistance rate from low to high, the top 10% score is set as 100, and the bottom 10% score is set as 0 |
| Data Source | National antimicrobial resistance (AMR) surveillance systems coordination capacity (who.int); PAHO/WHO Data - 3. Resistance percentages for selected pathogens; Surveillance Atlas of Infectious Diseases (europa.eu); Australian group on antimicrobial resistance - AGAR Reports; China Antimicrobial Resistance Surveillance System (<http://www.carss.cn/sys/Htmls/dist/index.html#/>) ; |

| Indicator Title | Third-generation β-lactams-resistent *Klebsiella pneumoniae* |
| --- | --- |
| Indicator code | 5.3.2 |
| Raw Data Title | Third-generationβ-lactams-resistent *Klebsiella pneumoniae* |
| Raw Data Abbreviation | BR-KPN |
| Value Range | (0, 97.40%) |
| Years Available | 2020 |
| Countries Available | 88 |
| Data Calculation/score Rule | In order of the resistance rate from low to high, the top 10% score is set as 100, and the bottom 10% score is set as 0 |
| Data Source | National antimicrobial resistance (AMR) surveillance systems coordination capacity (who.int); PAHO/WHO Data - 3. Resistance percentages for selected pathogens; Surveillance Atlas of Infectious Diseases (europa.eu); Australian group on antimicrobial resistance - AGAR Reports; China Antimicrobial Resistance Surveillance System (<http://www.carss.cn/sys/Htmls/dist/index.html#/>) ; |

| Indicator Title | Third-generation β-lactams-resistent *Escherichia coli* |
| --- | --- |
| Indicator code | 5.3.3 |
| Raw Data Title | Third-generationβ-lactams-resistent *Escherichia coli* |
| Raw Data Abbreviation | BR-ECO |
| Value Range | (5.8%, 100%) |
| Years Available | 2020 |
| Countries Available | 77 |
| Data Calculation/score Rule | In order of the resistance rate from low to high, the top 10% score is set as 100, and the bottom 10% score is set as 0 |
| Data Source | National antimicrobial resistance (AMR) surveillance systems coordination capacity (who.int); Surveillance Atlas of Infectious Diseases (europa.eu); Australian group on antimicrobial resistance - AGAR Reports; China Antimicrobial Resistance Surveillance System (<http://www.carss.cn/sys/Htmls/dist/index.html#/>) ; |

| Indicator Title | Third-generation β-lactams-resistent *Streptococcus pneumoniae* |
| --- | --- |
| Indicator code | 5.3.4 |
| Raw Data Title | Third-generationβ-lactams-resistent *Streptococcus pneumoniae* |
| Raw Data Abbreviation | BR-SPN |
| Value Range | (0, 64.71%) |
| Years Available | 2020 |
| Countries Available | 54 |
| Data Calculation/score Rule | In order of the resistance rate from low to high, the top 10% score is set as 100, and the bottom 10% score is set as 0 |
| Data Source | National antimicrobial resistance (AMR) surveillance systems coordination capacity (who.int); Surveillance Atlas of Infectious Diseases (europa.eu); Australian group on antimicrobial resistance - AGAR Reports; China Antimicrobial Resistance Surveillance System (<http://www.carss.cn/sys/Htmls/dist/index.html#/>) ; |

| Indicator Title | Third-generation β-lactams-resistent *Pseudomonas aeruginosa* |
| --- | --- |
| Indicator code | 5.3.5 |
| Raw Data Title | Third-generationβ-lactams-resistent *Pseudomonas aeruginosa* |
| Raw Data Abbreviation | BR-PAE |
| Value Range | (2.90%, 70.70%) |
| Years Available | 2020 |
| Countries Available | 41 |
| Data Calculation/score Rule | In order of the resistance rate from low to high, the top 10% score is set as 100, and the bottom 10% score is set as 0 |
| Data Source | PAHO/WHO Data - 3. Resistance percentages for selected pathogens; Surveillance Atlas of Infectious Diseases (europa.eu); Australian group on antimicrobial resistance - AGAR Reports; |

| Indicator Title | Macrolides-resistent *Streptococcus pneumoniae* |
| --- | --- |
| Indicator code | 5.4.1 |
| Raw Data Title | Macrolides-resistent *Streptococcus pneumoniae* |
| Raw Data Abbreviation | MR-SPN |
| Value Range | (3.50%, 96.00%) |
| Years Available | 2020 |
| Countries Available | 29 |
| Data Calculation/score Rule | In order of the resistance rate from low to high, the top 10% score is set as 100, and the bottom 10% score is set as 0 |
| Data Source | Surveillance Atlas of Infectious Diseases (europa.eu); China Antimicrobial Resistance Surveillance System (<http://www.carss.cn/sys/Htmls/dist/index.html#/>) ; |

| Indicator Title | Aminoglycosides-resistent *Klebsiella pneumoniae* |
| --- | --- |
| Indicator code | 5.5.1 |
| Raw Data Title | Aminoglycosides-resistent *Klebsiella pneumoniae* |
| Raw Data Abbreviation | AR-KPN |
| Value Range | (0, 75%) |
| Years Available | 2020 |
| Countries Available | 43 |
| Data Calculation/score Rule | In order of the resistance rate from low to high, the top 10% score is set as 100, and the bottom 10% score is set as 0 |
| Data Source | PAHO/WHO Data - 3. Resistance percentages for selected pathogens; Surveillance Atlas of Infectious Diseases (europa.eu); Australian group on antimicrobial resistance - AGAR Reports; |

| Indicator Title | Aminoglycosides-resistent *Acinetobacter baumannii* |
| --- | --- |
| Indicator code | 5.5.2 |
| Raw Data Title | Aminoglycosides-resistent *Acinetobacter baumannii* |
| Raw Data Abbreviation | AR-ABA |
| Value Range | (0, 97.10%) |
| Years Available | 2020 |
| Countries Available | 73 |
| Data Calculation/score Rule | In order of the resistance rate from low to high, the top 10% score is set as 100, and the bottom 10% score is set as 0 |
| Data Source | National antimicrobial resistance (AMR) surveillance systems coordination capacity (who.int); PAHO/WHO Data - 3. Resistance percentages for selected pathogens; Surveillance Atlas of Infectious Diseases (europa.eu); Australian group on antimicrobial resistance - AGAR Reports; |

| Indicator Title | Quinolone-resistent *Klebsiella pneumoniae* |
| --- | --- |
| Indicator code | 5.6.1 |
| Raw Data Title | Quinolone-resistent *Klebsiella pneumoniae* |
| Raw Data Abbreviation | QNR-KPN |
| Value Range | (0, 90.57%) |
| Years Available | 2020 |
| Countries Available | 83 |
| Data Calculation/score Rule | In order of the resistance rate from low to high, the top 10% score is set as 100, and the bottom 10% score is set as 0 |
| Data Source | National antimicrobial resistance (AMR) surveillance systems coordination capacity (who.int); PAHO/WHO Data - 3. Resistance percentages for selected pathogens; Surveillance Atlas of Infectious Diseases (europa.eu); Australian group on antimicrobial resistance - AGAR Reports; |

| Indicator Title | Quinolone-resistent *Escherichia coli* |
| --- | --- |
| Indicator code | 5.6.2 |
| Raw Data Title | Quinolone-resistent *Escherichia coli* |
| Raw Data Abbreviation | QNR-ECO |
| Value Range | (10.0%, 81.64%) |
| Years Available | 2020 |
| Countries Available | 75 |
| Data Calculation/score Rule | In order of the resistance rate from low to high, the top 10% score is set as 100, and the bottom 10% score is set as 0 |
| Data Source | National antimicrobial resistance (AMR) surveillance systems coordination capacity (who.int); Surveillance Atlas of Infectious Diseases (europa.eu); Australian group on antimicrobial resistance - AGAR Reports; China Antimicrobial Resistance Surveillance System (<http://www.carss.cn/sys/Htmls/dist/index.html#/>) ; |

| Indicator Title | Quinolone-resistent *Acinetobacter baumannii* |
| --- | --- |
| Indicator code | 5.6.3 |
| Raw Data Title | Quinolone-resistent *Acinetobacter baumannii* |
| Raw Data Abbreviation | QNR-ABA |
| Value Range | (0, 98.2%) |
| Years Available | 2020 |
| Countries Available | 26 |
| Data Calculation/score Rule | In order of the resistance rate from low to high, the top 10% score is set as 100, and the bottom 10% score is set as 0 |
| Data Source | Surveillance Atlas of Infectious Diseases (europa.eu); Australian group on antimicrobial resistance - AGAR Reports; |
